# mTORC1 underlies age‐related muscle fiber damage and loss by inducing oxidative stress and catabolism

**DOI:** 10.1111/acel.12943

**Published:** 2019-03-29

**Authors:** Huibin Tang, Ken Inoki, Susan V. Brooks, Hideki Okazawa, Myung Lee, Junying Wang, Michael Kim, Catherine L. Kennedy, Peter C. D. Macpherson, Xuhuai Ji, Sabrina Van Roekel, Danielle A. Fraga, Kun Wang, Jinguo Zhu, Yoyo Wang, Zelton D. Sharp, Richard A. Miller, Thomas A. Rando, Daniel Goldman, Kun‐Liang Guan, Joseph B. Shrager

**Affiliations:** ^1^ Division of Thoracic Surgery, Department of Cardiothoracic Surgery Stanford University School of Medicine Stanford California; ^2^ VA Palo Alto Healthcare System Palo Alto California; ^3^ Life Science Institute University of Michigan Ann Arbor Michigan; ^4^ Department of Molecular and Integrative Physiology University of Michigan Ann Arbor Michigan; ^5^ Department of Pharmacology and Moores Cancer Center University of California San Diego La Jolla California; ^6^ Molecular and Behavioral Neuroscience Institute and Department of Biological Chemistry University of Michigan Ann Arbor Michigan; ^7^ Human Immune Monitoring Center, Stanford University School of Medicine Stanford California; ^8^ Department of Pathology and Geriatrics Center University of Michigan Ann Arbor Michigan; ^9^ Department of Molecular Medicine University of Texas Health Science Center at San Antonio San Antonio Texas; ^10^ Paul F. Glenn Laboratories for the Biology of Aging and Department of Neurology and Neurological Sciences Stanford University School of Medicine Stanford California; ^11^ Present address: The Department of Thoracic Surgery Third Affiliated Hospital of Kunming Medical University Kunming China; ^12^ Present address: Department of Cardiothoracic Surgery Guangxi International Zhuang Hospital of GuangXi University of Chinese Medicine NanNing China

**Keywords:** aging, mTORC1, oxidative stress, signal transduction, skeletal muscle

## Abstract

Aging leads to skeletal muscle atrophy (i.e., sarcopenia), and muscle fiber loss is a critical component of this process. The mechanisms underlying these age‐related changes, however, remain unclear. We show here that mTORC1 signaling is activated in a subset of skeletal muscle fibers in aging mouse and human, colocalized with fiber damage. Activation of mTORC1 in TSC1 knockout mouse muscle fibers increases the content of morphologically abnormal mitochondria and causes progressive oxidative stress, fiber damage, and fiber loss over the lifespan. Transcriptomic profiling reveals that mTORC1's activation increases the expression of growth differentiation factors (GDF3, 5, and 15), and of genes involved in mitochondrial oxidative stress and catabolism. We show that increased GDF15 is sufficient to induce oxidative stress and catabolic changes, and that mTORC1 increases the expression of GDF15 via phosphorylation of STAT3. Inhibition of mTORC1 in aging mouse decreases the expression of GDFs and STAT3's phosphorylation in skeletal muscle, reducing oxidative stress and muscle fiber damage and loss. Thus, chronically increased mTORC1 activity contributes to age‐related muscle atrophy, and GDF signaling is a proposed mechanism.

## 1 INTRODUCTION

Age‐dependent loss of skeletal muscle mass and function, that is, sarcopenia, is an increasingly significant public health concern as average life expectancy increases. Degeneration in muscle structure and function during aging increases frailty, disability, metabolic disorders, and mortality in the elderly population (Evans et al., 2010). It is thus central to the aging process. Numerous changes in aging skeletal muscle have been reported, including muscle fiber loss and induction of pathological regeneration reflected by centrally located myonuclei (Larsson, Sjödin, & Karlsson, 1978; Marzetti, Lees, Wohlgemuth, & Leeuwenburgh, 2009; Narici & Maffulli, 2010). Mitochondria are significantly altered in aged skeletal muscle, where they are more abundant but with reduced function (Short et al., 2005), morphologically abnormal, and more variable in size, with the appearance of enlarged and giant mitochondria (Arking, 2006; Demontis, Piccirillo, Goldberg, & Perrimon, 2013; Miller et al., 2008; Murakoshi, Osamura, & Watanabe, 1985; Tandler & Hoppel, 1986). Dysfunctional mitochondria in muscle fibers may underlie apoptosis and necrosis, which likely plays a considerable role in aging muscle fiber loss (Cheema, Herbst, McKenzie, & Aiken, 2015). All of these changes likely result in increased muscle fatigability and decreased force generation. The etiology of these age‐dependent deleterious changes and ultimate fiber loss in skeletal muscle, however, remains largely unclear.

The mechanistic target of rapamycin, mTOR, is an evolutionarily conserved serine–threonine protein kinase which is generally thought to promote various anabolic cellular processes in response to growth factors, nutrients, and stress (Inoki, Corradetti, & Guan, 2005; Sabatini, 2017; Saxton & Sabatini, 2017). mTOR exists in two distinct complexes, mTORC1 and mTORC2, and the former is directly and sensitively inhibited by rapamycin. The tuberous sclerosis complex (TSC) 1 and TSC2 tumor suppressors are major negative regulators of mTORC1. Mutation in either TSC1 or TSC2 leads to constitutive activation of mTORC1. Activated mTORC1 phosphorylates downstream targets, for example, S6 kinase and eIF4E binding proteins, and S6 kinase further phosphorylates ribosomal S6 protein, facilitating protein synthesis. The level of phosphorylated S6 protein is often used to probe the activity of mTORC1 signaling. mTORC1 regulates developmental muscle growth, and Akt‐dependent activation of mTORC1 is positively associated with muscle mass. For instance, inhibition of mTORC1 through either deletion of the mTORC1 component, raptor (Bentzinger et al., 2008), or overexpression of the mTORC1 suppressor, TSC1 (Wan et al., 2006), causes muscle atrophy. In contrast, activation of mTORC1 by overexpression of constitutively active Akt, an mTORC1 upstream activator, induces muscle hypertrophy (Bodine et al., 2001). However, Akt‐independent activation of mTORC1 leads to protein degradation and muscle atrophy (Bentzinger et al., 2013; Castets et al., 2013; Tang et al., 2014), likely through feedback inhibition of Akt and the consequent activation of FoxO (Tang et al., 2014). Beyond this dual function of mTORC1 in proteostasis, activated mTORC1 also regulates metabolism by induction of FGF21 (Cornu et al., 2014; Guridi et al., 2015) and regulates mitochondrial function, including oxygen consumption (Schieke et al., 2006) and oxidative function (Cunningham et al., 2007), in cultured cells. It was reported that the activities of mTORC1 and its downstream S6 kinase are induced in aged skeletal muscle (Baar, Carbajal, Ong, & Lamming, 2016; Houtkooper et al., 2011; Kimball & Jefferson, 2004; Markofski et al., 2015). However, the downstream effects of the activated mTORC1 in aging skeletal muscle remain unknown.

Growth differentiation factors (GDFs) are subgroup members in the TGF‐β (transforming growth factor beta) superfamily that function predominantly in regulating development. For example, GDF3 and GDF5 are involved in bone formation in early embryonic development (Buxton, Edwards, Archer, & Francis‐West, 2001; Levine & Brivanlou, 2006). GDF8/myostatin negatively regulates muscle growth (McPherron, Lawler, & Lee, 1997). GDF15, also known as macrophage inhibitory cytokine 1 (MIC‐1) or nonsteroidal anti‐inflammatory drug‐activated gene (NAG‐1), has a known role in regulating tissue injury (Zimmers et al., 2005) and lipolysis (Chrysovergis et al., 2014). GDF15 is also involved in mitochondrial diseases (Kalko et al., 2014; Montero et al., 2016; Yatsuga et al., 2015) and all‐cause mortality (Wiklund et al., 2010), regulates appetite, weight loss, and cancer‐related anorexia (Breit et al., 2011; Johnen et al., 2007; Lerner et al., 2015; Tsai, Lin, Brown, Salis, & Breit, 2016; Tsai et al., 2013, 2014; Villars, Pietra, Giuliano, Lutz, & Riediger, 2017). However, neither the functional role of GDFs (beyond GDF8) in skeletal muscle, nor their regulation, has been described.

In the current study, we investigate whether mTORC1 activity is involved in the phenotypic changes associated with muscle aging, and if so, what underlying mechanisms are implicated. We report here that mTORC1 activity is induced in a subset of aging skeletal muscle fibers in both human and mouse, and that these mTORC1‐activated fibers often exhibit degenerative changes. We further demonstrate in a transgenic mouse model that activated mTORC1 is sufficient to induce oxidative stress in muscle, and myofiber damage and loss. Our work establishes that activated mTORC1 transcriptionally upregulates GDFs, including GDF3, 5, and 15, in addition to inducing FoxO family members as previously reported, and that the GDFs are sufficient to induce catabolic changes in skeletal muscle. mTORC1 activity induces GDF15 expression via upregulating the phosphorylation of STAT3. Inhibition of mTORC1 with rapamycin treatment relieves oxidative stress and reduces muscle fiber loss in old mice.

## 2 RESULTS

### 2.1 mTORC1 is activated in aged skeletal muscle fibers of both mouse and human, and its overactivity co‐localizes with degenerative changes in muscle fibers

**Figure 1:**
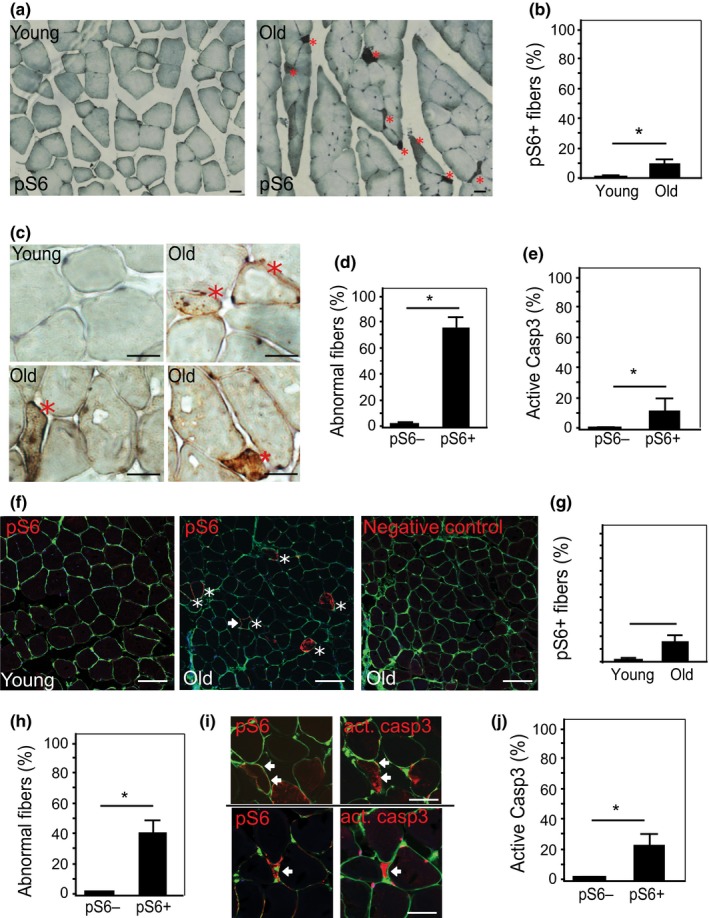
Activation of mTORC1 in aged muscle is colocalized with degenerative changes in both mouse and human skeletal muscle. (a and b) Immunostaining on young (2.5 months[m]) and old (30 m) mouse skeletal muscles with antibody against pS6 protein, visualized by DAB (Diaminobenzidine) (5x, 40x). pS6 + fibers (brown, indicated by *) appear mainly in old muscle, scattered, with angular shapes and smaller diameter. Nuclei were stained with hematoxylin (blue). Scale bar: 20 µm. (c) Increased pS6 + fibers in old mouse TA muscles. ~300 fibers/muscle were counted, *n* = 3 mice, *p < 0.05. (d) Quantification of the abnormal fibers and pS6 staining. In aged mice, the percentage of abnormal fibers is significantly higher among pS6 + fibers than that pS6‐ fibers. ~150 fibers/mouse, *n* = 3 mice, *p < 0.05. (e) Increased caspase 3 activity in pS6 + fiber in old muscles. Consecutive sections were with anti‐pS6 and anticleaved caspase 3 (active casp3) antibodies. Quantitation was performed on ~100 fibers/mouse, *n* = 3 mice, *p < 0.05. (f) Immunofluorescence staining with anti‐pS6 in young (23 years, left pane) and old (78 years, middle pane) human latissimus dorsi muscles. pS6 + fibers (red) appear in old muscle, and some of these demonstrate the same angular shape and smaller diameter (arrow) seen in pS6 + fibers in old mouse muscle. Cell membrane was stained with wheat germ agglutinin (WGA, green). Scale bar: 50 µm. Negative control (right panel): absence of primary antibody. (g and h) Quantitative results show the higher percentage of pS6 + fibers in old muscle (g) and the higher percentage of fibers with abnormal phenotypes (h) (smaller size and/or irregular shape) in pS6+ vs. pS6− fibers. 200 fibers/muscle, young: *n* = 6 muscles; old: *n* = 7 muscles; *p < 0.05. (i) pS6+ muscle fibers stain positively for activated (cleaved) caspase 3. Consecutive muscle sections were stained with antibodies against pS6 and cleaved caspase 3 (act. Casp3), respectively. Cell membrane was stained with WGA. Matching fibers are indicated with arrows. Scale bar 25 µm. (j) Quantitative result shows a higher percentage of positive staining for activated caspase in pS6+ than pS6− muscle fibers in old muscles. ~200 fibers/muscle, counted in 7 muscles, *p < 0.05

To explore the functional role of the mTORC1 signaling pathway in aged muscle, we first examined mTORC1 activity by probing the phosphorylation of its downstream target protein S6 (pS6). Immunostaining revealed increased pS6‐stained fibers scattered within the muscles of aged *mice* (30 month, 11% ± 2%) vs. their young counterparts (2 months, 0.3%, *p* < 0.05; Figure 1a–c). The activation of mTORC1 in muscle fibers was also detected at 12 and 24 months of age, indicating a progressive induction in aging muscle over time (Supporting Information Figure S1a,b). Interestingly, the majority of the pS6‐positive fibers in the aged muscle are phenotypically abnormal, demonstrating changes including irregular shape and visibly smaller CSA (Figure 1b and Supporting Information Figure S1c). Among the pS6‐positive fibers at 30 months old, 76.2% ± 7% exhibit reduced CSA and/or angular shape, which is significantly higher than the rate in the pS6‐negative fibers (2.02% ± 1%, *p* < 0.05; Figure 1d). In addition, 11% of the pS6+ fibers show positive staining for activated caspase 3, comparing to 1% in pS6‐ fibers (Figure 1e).

Similarly, mTORC1 activity increases in *human* skeletal muscle fibers during aging. In a younger cohort (42 ± 12 years), human latissimus dorsi muscle exhibits rare pS6+ fibers (3% ± 1%). The pS6+ fiber percentage significantly increases (to 15% ± 4%, *p* < 0.05) in older latissimus (69 ± 6 years; Figure 1f,g), consistent with the increased mTORC1 activity in aged muscle that has been previously reported (Markofski et al., 2015). Approximately 40% of the pS6+ human muscle fibers show abnormal phenotypes, including angular shape and reduced size (Figure 1h). Twenty‐two percent of the human pS6+ fibers express the apoptotic marker cleaved caspase 3 in the cytoplasm, compared to 2% in the pS6− fibers (Figure 1i,j).

Taken together, the occurrence of these abnormal degenerative phenotypes in skeletal muscle fibers from older mice and humans that have increased pS6 staining indicates that elevated mTORC1 activity in aging muscle fibers is associated with muscle fiber damage. These degenerative processes, resulted in part from the activation of apoptotic signaling, contribute to the age‐related muscle atrophy.

### 2.2 Chronic activation of mTORC1 is sufficient to cause progressive muscle atrophy, fiber damage, fiber death, and muscle weakness

**Figure 2:**
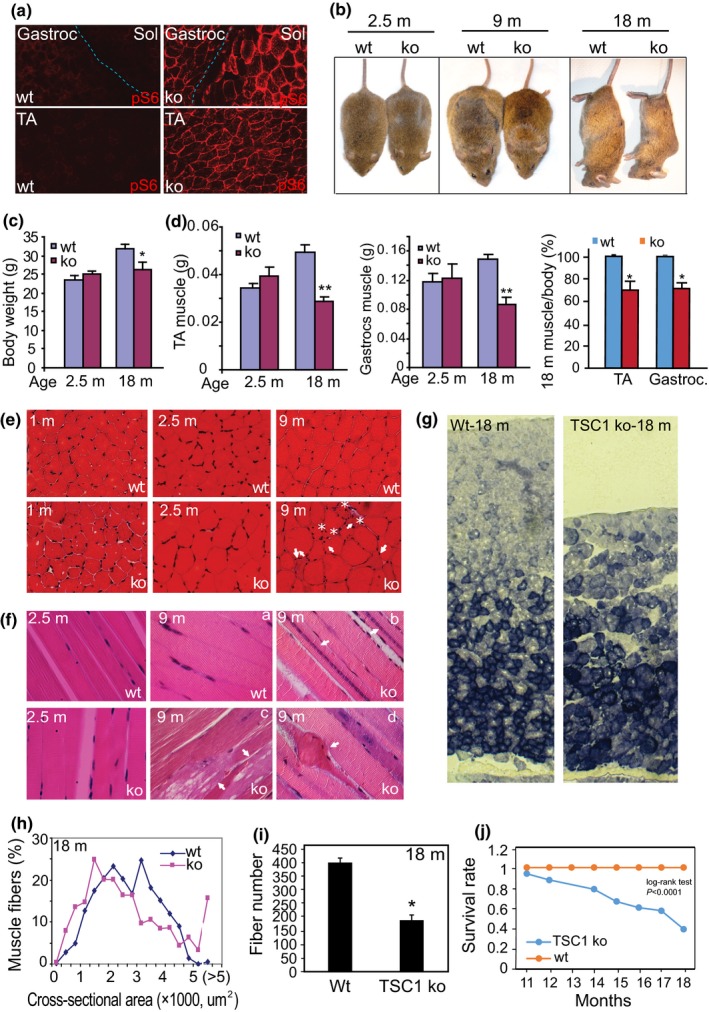
Muscle‐specific activation of mTORC1 in mice leads to progressive muscle fiber damage, fiber death, and loss of muscle mass. (a) Immunofluorescence staining with anti‐pS6 antibody in muscle harvested from TSC ko and wt mice at 2.5‐month‐old. pS6+ staining indicates that the mTORC1 signaling pathway is activated in both slow (Sol: soleus muscle) and fast‐twitch muscles (TA: tibialis anterior; Gastroc: gastrocnemius muscle) of TSC1 ko animals. (b and c) Age‐dependent reduction in body size and weight in TSC1 ko mice. The body weight of TSC1 ko mice is reduced at 18 months (18 m) relative to wt controls. *n* = 4, *p < 0.05. Note that the old (18 m) TSC1 ko mice develop a hunched back. (d) Age‐dependent reduction in muscle mass in TA and gastrocnemius muscles of TSC ko mice vs. wt controls. *n* = 4, **p < 0.001 at 18 months. Note, the muscle/body weight ratio is also significantly reduced in TSC ko mice. (e) Histology of mTORC1‐activated muscles and their wt controls. Cross sections of TA muscle at 1 m, 2.5 m, and 9 m (HE staining). Note that some fibers in TSC1 ko muscle at 9 m are shrunken (arrows and asterisks) and/or have centrally located nuclei (arrows). (f) Longitudinal sections of TA muscle at 2.5 m and 9 m (HE staining). Note that in 9 m TSC1 ko muscle, some fibers contain nuclear chains (arrow, b), cytoplasmic vacuolation (arrow, c), and death (arrow, d). (g) Preferential loss of glycolytic muscle fibers in response to mTORC1 activation. SDH enzymatic activity was assayed on sections from the lateral head of the gastrocnemius muscle. Note that predominantly glycolytic (SDH‐) fibers are lost in TSC1 ko muscle at 18 m. (h) Distribution of the cross‐sectional area (CSA) of the muscle fibers from wt and TSC1 ko mice at 18 months. Note, the leftward shift of the distribution curve in the ko indicating decreased CSA. (i) Reduced fiber number in TSC1 ko muscle at 18 m. Cross sections of the lateral head of gastrocnemius muscle were taken, and fiber numbers in a 10x image from an identical location (~1/4 of the lateral head) were counted, *n* = 4 mice, *p < 0.01. (j) Reduced lifespan in the TSC1 ko mice, compared to wt controls. The comparison of the survival rate between wt and ko mice was examined by the log‐rank test (implemented in STATA). The log‐rank *p* value is *p* < 0.0001. The median lifespan of the TSC1 ko mice was 18 months. Detail in supporting information Figure S4e

In order to determine whether mTORC1 activation is the cause of the degenerative changes in aging skeletal muscle, we first tested if mTORC1 activation is sufficient to induce these degenerative changes. We examined a mouse model in which the mTORC1 inhibitor, TSC1, is genetically deleted in a muscle‐specific manner, leading to the activation of mTORC1 in skeletal muscle, as described previously (Bentzinger et al., 2013; Castets et al., 2013; Tang et al., 2014). The knockout of TSC1 (TSC1 ko) by breeding floxed TSC1 mice with MCK‐cre mice was confirmed by RT–PCR (Supporting Information Figure S2), and the activation of mTORC1 was validated by the positive staining of pS6 in various muscles—gastrocnemius, TA, and soleus (Figure 2a and Supporting Information Figure S3). TSC1 ko mice displayed no discernible phenotypic changes or growth defects at 2.5 months of age, with similar body size/weight and muscle weight as wt mice (Figure 2b–d). However, as they aged, the TSC1 ko mice became physically smaller compared to their wt counterparts. At 18 months of age, the TSC1 ko mice have a 20% lower body weight than wt and exhibit kyphosis often seen with severe illness and advanced age (Figure 2b,c). The weight of the gastrocnemius and TA muscles were dramatically reduced at this age to approximately 60% of their wt controls (Figure 2c). The relative muscle weight (muscle weight vs. body weight) in TSC1 ko mice was also significantly reduced to ~72% of the wt controls, indicating that muscle wasting is a primary change following mTORC1 activation (Figure 2d).

The histological changes in mTORC1‐activated skeletal muscle are also progressive. Cross sections of muscle fibers appear normal in the young TSC1 ko mice (1 month), but the CSA of the muscle fibers starts to enlarge at 2.5 months of age (Figures 2e and 5a). By 9 months of age, TSC1 ko muscle fibers have become visibly heterogeneous, with 17% ± 3% shrunken fibers containing central nuclei interspersed among enlarged fibers (Figure 2e), whereas there are no visible abnormal fibers in the wt muscle at this age. Longitudinal sections of 9‐month‐old TSC1 ko muscles reveal that some fibers contain long chains of myonuclei aligned head‐to‐tail in the center of the muscle fibers; these myonuclear chains are never observed in wt myofibers (Figure 2f–b) and indicate that the affected fibers are actively regenerating due to damage. In addition, cellular vacuolation (Figure 2f–c) and death of individual muscle fibers (Figure 2f–d) are also seen in the TSC1 ko muscles but not in wt control mice at that age (Figure 2f–a).

We stained the muscle sections for succinate dehydrogenase (SDH) activity, which identifies slow oxidative fibers (SDH+) enriched in mitochondria. Compared to wt mice, TSC1 ko mice exhibit a gradual loss of fast glycolytic fibers (SDH‐) as they age to 18 months (Figure 2g), indicating that chronic mTORC1 hyperactivity may lead to a preferential loss of glycolytic fibers. Although at 2.5 months of age the muscle fibers are generally larger in TSC1 ko fibers compared to wt, they become more variable at 18 months of age, when most muscle fibers are smaller than wt; though with 16% ± 3% of the fibers larger than wt (more than twice the mean size of wt fibers; Figure 2h). TSC1 ko muscle is thus composed of widely varied muscle fiber sizes at old age. These phenotypes imply that chronic mTORC1 activation leads to the muscle damage and atrophy after an initial phase of hypertrophy. Muscle fiber number in TSC1 ko mice at 18 months old is significantly reduced. The lateral head of gastrocnemius muscle, for example, loses approximately 50% of its muscle fibers in 18‐month TSC1 ko mice (Figure 2i).

The exercise capacity of the TSC1 ko mice is also compromised, with significant reduction of their maximum running time on treadmill at 9 months of age (Supporting Information Figure S4). We also observed that both the maximal and specific isometric contractile forces of isolated TSC1 ko muscles (both EDL and Soleus) are reduced compared to wt controls (Supporting Information Figure S4 and Table S1). In addition, the lifespan of the TSC1 ko mice is significantly shorter (*p* < 0.0001), with only about 39% of TSC1 ko mice surviving to 18 months (Figure 2j and Supporting Information Figure S4). This may be due to whole‐body metabolic changes driven by mTORC1 activation (Guridi et al., 2015), respiratory failure secondary to compromised respiratory muscles, or other incompletely evaluated phenotypes of accelerated aging associated with mTORC1 and/or GDF15.

### 2.3 Chronic activation of mTORC1 induces abnormal mitochondria in skeletal muscle, accompanied by oxidative stress and the activation of caspase 3

**Figure 3:**
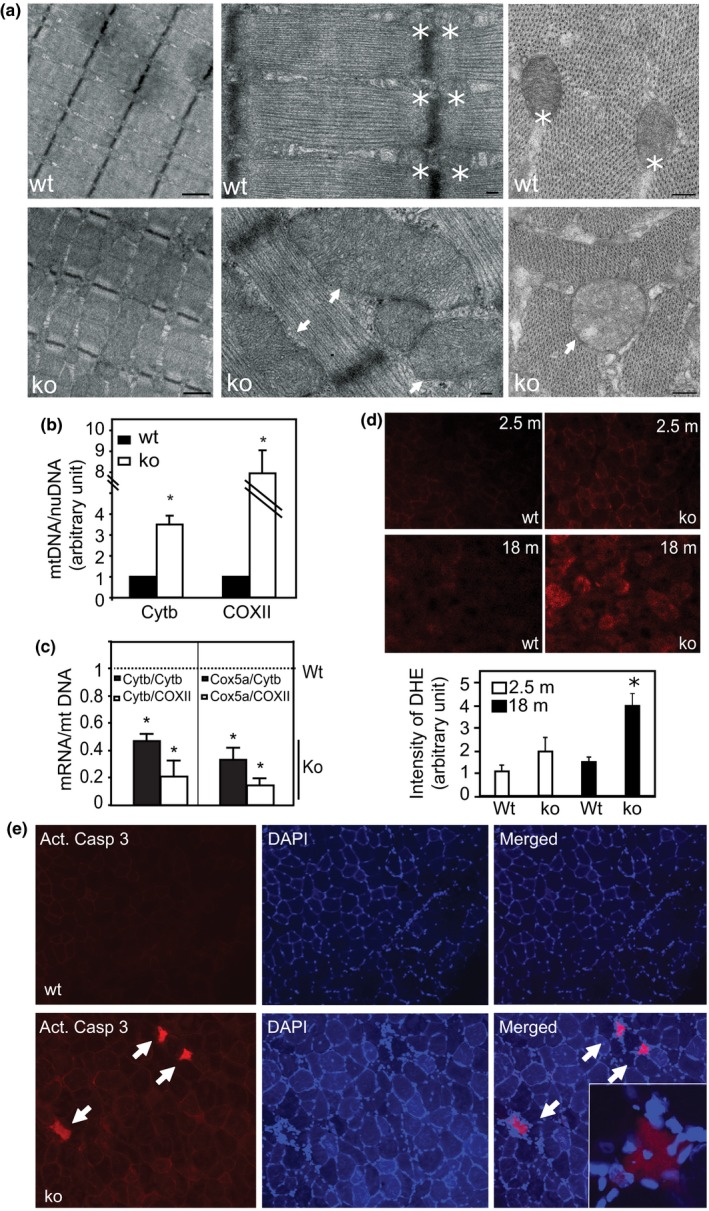
Activated mTORC1 leads to abnormal mitochondrial proliferation, oxidative stress, and muscle fiber apoptosis.(a) Electron microscopic view of muscle tissue to demonstrate sarcomeres and mitochondria. Left panel: The structure and alignment of the sarcomeres are normal in 7 m TSC1 ko muscles (scale bar is 1 µm). Middle panel: The TSC1 ko muscle has normal myofilaments, but mitochondria are increased to sometimes enormous size (arrows). Normal mitochondria in wt muscle are indicated with asterisks (*). Scale bar: 200 nm. Right panel: cross‐sectional view of sarcomere. Normal density and distribution of myofilaments, but mitochondria (arrows) in TSC1 ko muscle become round and lose internal cristae density vs. wt mitochondria (*). (b) Mitochondrial content shown by relative DNA ratio of mitochondrial vs. myonuclear genes. Cytb, cytochrome b; COXII, cytochrome C oxidase subunit II. *n* = 4, *p < 0.05. (c) The reduction in the relative levels of the mRNAs of mitochondrial function‐associated genes vs. mitochondrial content (DNA) in TSC1 ko muscle. The mRNA expression of the mitochondrial function‐associated genes (Cytb and Cox5a) was quantified and normalized to the mitochondrial DNA levels represented here by Cytb DNA and COXII DNA, respectively. mRNA and DNA levels are both quantified by real‐time PCR. *n* = 4, *asterisk denotes *p* < 0.05 for the comparison of mRNA/mt DNA ratios between the ko and the Wt control, which is set as 1 (unity). (d) Increased oxidative stress in 18 m TSC1 ko muscle. DHE staining (red) was performed on muscle cryosections. Note that reactive oxygen species (ROS) is detected at a higher level in TSC1 ko muscle at 18 m, shown by intensity quantitation in the below bar graph. *n* = 3, *p < 0.05. (e) Induced apoptosis in TSC1 ko muscle. Immunostaining with anticleaved caspase‐3, counterstained with DAPI on 9 m muscle. Note, caspase‐3^+^ fibers (red) are present in TSC1 ko muscle, and have central nuclei (inset)

We also examined the ultrastructure of the muscles by transmission electron microscopy (TEM) with particular attention to mitochondrial changes. In TSC1 ko muscle (~7 months old), sarcomere length, structure, and organization, as well as the alignment of sarcomeres and myofilaments, appear similar to wt at this age. Mitochondria, however, are significantly enlarged vs. wt, and they are no longer restricted to the small area flanking the Z band, where mitochondria are normally located. Rather, the mitochondria became gigantic, often spanning the full length of the sarcomere. Similarly, muscle fiber cross sections reveal the density of TSC1 ko myofilaments to be normal, but the mitochondria are round, with reduced internal cristae density, indicating mitochondrial matrix swelling (Figure 3a).

The mitochondrial content is significantly increased in TSC1 ko muscle, based upon the ratio of mitochondrial genes (COXII/cytochrome C oxidase II and Cytob/Cytochrome b), relative to the myonuclear gene beta‐actin (Figure 3b). This increase in mitochondrial content may result from both accelerated mitochondrial biogenesis and inhibition of autophagy, driven by the hyperactive mTORC1 in TSC1 ko muscle. However, these enlarged and proliferating mitochondria appear to be dysfunctional, since the relative RNA expression levels of genes reflective of mitochondrial function such as COX5a and cytochrome b, relative to mitochondrial DNA/content, are significantly reduced (Figure 3c).

We suspected that the presence of abnormal and functionally compromised mitochondria in TSC1 ko muscles would lead to mitochondrial oxidative stress (MOS). Indeed, reactive oxygen species (ROS) are increasingly accumulated in TSC1 ko muscle with age, as demonstrated by dihydroethidium (DHE) staining (Figure 3d). Further, immunoreactivity for the apoptotic marker cleaved caspase‐3 is induced in mTORC1‐activated muscle fibers, accompanied by centrally located myonuclei (Figure 3e). Together, these data strongly imply that MOS‐associated intrinsic apoptosis is induced by chronic activation of mTORC1.

### 2.4 Activation of mTORC1 induces the expression of growth differentiation factors and genes/proteins involved in mitochondrial oxidative stress and apoptosis

**Figure 4:**
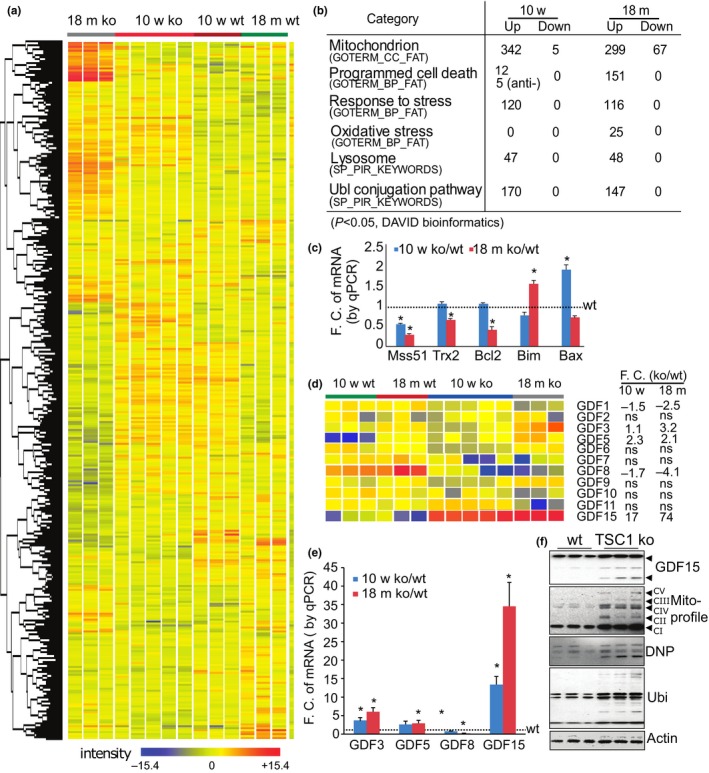
Activated mTORC1 alters the transcriptomic expression profile of genes involved in mitochondrial oxidative stress and apoptosis, as well as members of the GDF family. (a) Heatmap of the clustered transcriptomic profiles (*p* < 0.05) comparing wt and TSC1 ko muscles at 2.5 m and 18 m, analyzed by GeneSpring software. (b) Signaling mined by DAVID bioinformatics software. The number of significantly altered genes (*p* < 0.05) in each pathway/event is listed. (c) Quantitative PCR to validate the altered expression of genes related to mitochondria and oxidative stress. Gamma actin was used as normalization control (F.C, Fold change). Antioxidant and anti‐apoptotic genes (e.g., Mss51, trx2, and Bcl2) are decreased, whereas the apoptotic genes (e.g., Bim and Bax) are increased. *n* = 3, *p < 0.05 compared to wt controls. (d) The expression profile of GDF family members comparing wt and TSC ko at 2.5 and 18 months. GDF 15 is by far the most dramatically upregulated. ns: no significant change. (e) Quantitative PCR validating the altered expression of genes in the GDF family. Gamma actin was used as normalization control (F.C, Fold change). *n* = 3, *p < 0.05 compared to wt controls. (f) Protein expression profiles by Western blot show the induction of GDF15, mitochondrial components, protein oxidation, and polyubiquitination (ubi) in mTORC1‐activated muscle (18 m), actin as loading control. GDF15 blot: black arrow pointing to mature dimer of GDF15 (~25 kDa); asterisk pointing to immature intermediate GDF15 protein products. The presence of multiple bands of GDF15 is further explained and validated in Supporting Information Figures S5, S6

To begin to understand the molecular mechanisms underlying the phenotypic changes seen in the skeletal muscle of the mTORC1‐hyperactive TSC ko mice, we performed mRNA microarray. Muscle samples (gastrocnemius) from 2.5‐ and 18‐month‐old wt and TSC1 ko mice were harvested and subjected to Agilent single color mRNA microarray. Data were analyzed by GeneSpring and DAVID bioinformatics analysis software (Figure 4a). As summarized in Figure 4b, genes associated with mitochondria, stress response, and protein degradation pathways (e.g., lysosomal and ubiquitin–proteasome systems) are significantly upregulated in TSC1 ko muscles at both 2.5 and 18 months old. The genes involved in mitochondrial oxidative stress and programmed cell death are also dramatically induced, but primarily only at 18 months. This reveals a progressive induction of oxidative stress and apoptosis in skeletal muscle following chronic mTORC1 activation. The reduced expression of mitochondrial function‐associated gene (Mss51), antioxidant gene (Thioredoxin 2), and anti‐apoptotic genes (Bcl2), and the increased expression of pro‐apoptotic genes (Bim, Bax) were further validated by quantitative PCR (Figure 4c).

Considering all of the genes with upregulated expression, GDF15 is among the most strongly induced, and it is persistently increased from 2.5 to 18 months. Other members of the GDF family, such as GDF3 and 5, are also induced, but GDF8/myostatin is reduced (Figure 4d), and the remaining GDFs are not significant changed. These gene expression levels were also further validated by quantitative PCR (Figure 4e). The increased expression of GDF15 was also further confirmed at the protein level (Figure 4f). In addition, the expression of component proteins in the mitochondrial respiratory chain—such as the complex V component ATP5A, the complex III component UQCRC2, the complex IV component MTCO1, the complex II component SDHB, and the complex I component NDUFB8—is upregulated, suggesting that mTORC1 increases mitochondrial content. The oxidative stress marker—protein carbonylation, revealed by DNP (dinitrophenyl) levels—is also increased (Figure 4f). A measured increase in polyubiquitinated proteins is also consistent with the transcriptomic profiling that shows the induction of genes involved in ubiquitin–proteasome‐mediated protein degradation. Taken together, these molecular changes reveal that mTORC1 activation leads to MOS, protein degradation, and apoptosis—and the GDF family appears to be a likely participant in the regulation of these processes.

### 2.5 Inhibition of mTORC1 activity with rapamycin in TSC1 ko mice normalizes the molecular and phenotypic changes in skeletal muscles

**Figure 5:**
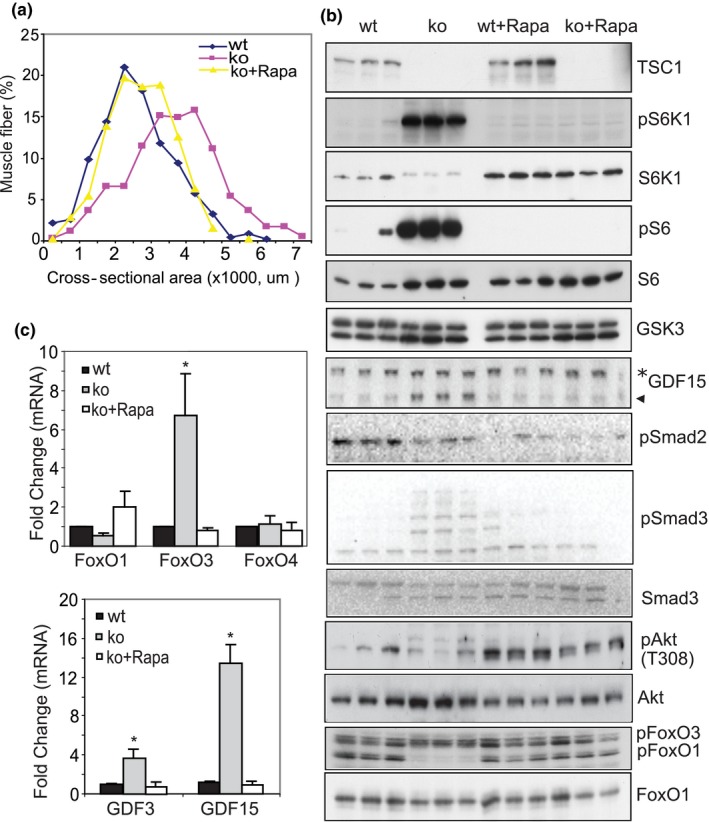
Inhibition of mTORC1 activity with rapamycin prevents the altered phenotype and molecular signaling in TSC1 ko muscle. (a) Distribution of fiber CSA in wt, TSC1 ko, and TSC ko + rapamycin. Muscle fiber enlargement seen in mTORC1 activated muscle (~3 m) is inhibited by 8 weeks of rapamycin treatment, beginning at 5 weeks old. (b) Molecular signaling that is altered in the ko mouse muscle is normalized with rapamycin to a wt‐like expression pattern. Western blot showing altered activities in the mTORC1‐S6K‐S6, GDF‐Smad, and Akt‐FoxO signaling pathways. Note that pS6K1 and pS6 levels, as well as GDF15 and pSmad3, are all dramatically increased in TSC1 ko mice, but rapamycin treatment recovers these changes. In contrast, decreased pAkt/Akt and pFoxO1/FoxO1 in TSC1 ko muscle is upregulated by rapamycin treatment. (c) The transcriptional upregulation of FoxO3 and GDF15 by activated mTORC1 is suppressed by rapamycin. mRNA levels were measured by quantitative PCR, and the level of gamma actin was used control. Fold changes vs. wt were shown. *n* = 3, *p < 0.05

To further investigate if the associated changes in TSC1 ko mice are due to mTORC1 hyperactivity, we treated TSC1 ko mice with rapamycin for 8 weeks beginning at 5 weeks of age. The main phenotypic change in the skeletal muscle of TSC1 ko mice at 13 weeks is enlarged fiber CSA (Figure 5a). We observed that inhibition of mTORC1 activity with rapamycin restores fiber CSA, which is increased in TSC1 ko muscle, back to that of wt (Figure 5a).

At the molecular level, rapamycin treatment normalized the altered mTORC1 signaling pathway by reducing the phosphorylation levels of S6K1 and S6 without affecting the protein levels of S6K1, S6, and GSK3. Inhibition of mTORC1 also led to the suppression of the elevated expression of GDF15 protein (Figure 5b). Interestingly, a downstream signaling mediator of the GDF family, Smad3, was significantly activated (phosphorylated) in TSC1 ko muscle, but this returned to baseline with inhibition of mTORC1 activity (Figure 5b). However, Smad2 phosphorylation was not significantly affected by mTORC1 activity and remained unchanged with rapamycin treatment. On the other hand, the suppressed Akt activity (pAkt/total Akt) in the TSC1 ko was recovered by rapamycin treatment, and the activation of FoxO, revealed by the level of phosphorylated FoxO1/total FoxO1, returned to baseline with rapamycin treatment (Figure 5b). The upregulated transcriptional expression of GDF15 and FoxO3 mRNAs was also completely normalized by rapamycin treatment (Figure 5c).

Thus, both the molecular and the phenotypic changes within skeletal muscle which are present in TSC1 ko mice are prevented by rapamycin treatment, indicating that the changes observed in TSC1 ko mice are due to the activation of mTORC1.

### 2.6 GDFs are sufficient to induce oxidative stress and catabolic activity in skeletal muscle

**Figure 6:**
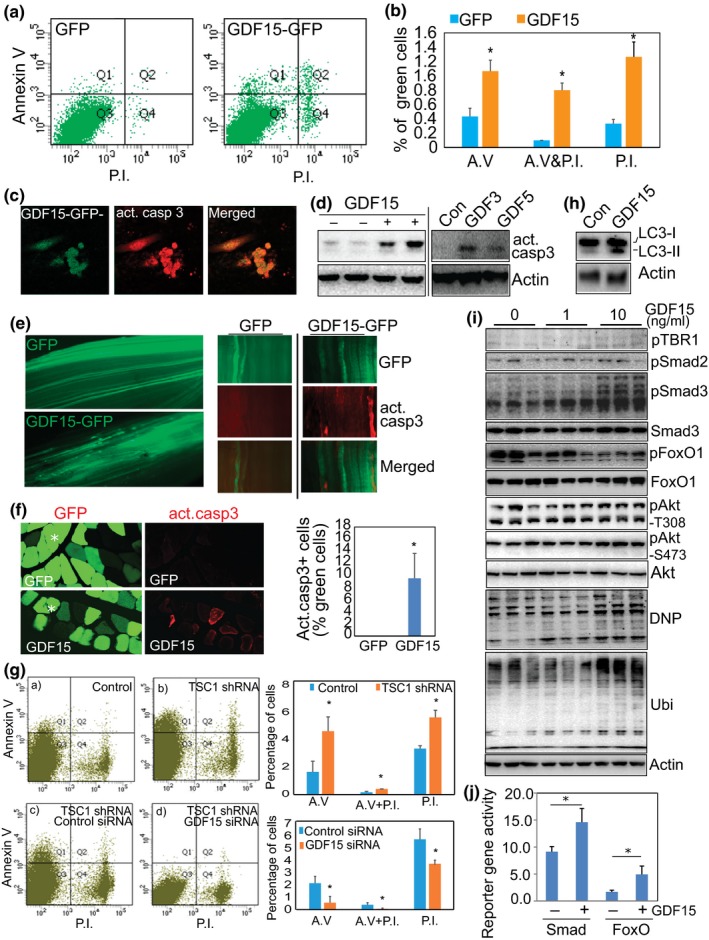
GDF is sufficient to induce apoptosis in muscle cells and is required for mTORC1‐induced apoptosis through regulation of Smad3, FoxO1, and oxidative stress (a) GDF15 induces apoptotic changes in cultured cells. C2C12 cells were transfected with GDF15‐GFP and control GFP plasmids. One day later, cells were harvested, stained with Annexin V and Propidium Iodide (PI), and analyzed by flow cytometry. Shown is the distribution of GFP+ and GDF15‐GFP+ cells. (b) Quantitative data of the distribution of the positively stained cells in the different quadrants. *n* = 3, *p < 0.05. (c) GDF15‐transfected cells show positive staining of cleaved caspase 3. Immunostaining was performed with anticleaved caspase 3 (red), 3 days after transfection of GDF15‐GFP (green) into C2C12 cells. (d) GDF increases the protein level of activated/cleaved caspase 3. Recombinant GDF3, 5, and 15 (10 ng/ml) was used to treat differentiated C2C12 myotubes for 4 days, followed by detection of cleaved caspase 3 by Western blot analysis. (e and f) GDF15 activates caspase 3 in vivo. TA muscle was electroporated with GDF15‐GFP or control GFP plasmids (20 μg/injection). Fourteen days later, TA muscles were harvested and fixed with paraformaldehyde for whole‐mount staining (e) or cross‐sectional staining (f) with anticleaved caspase 3. Note that some GDF15+ fibers stain positively for cleaved/activated caspase 3 (red). (g) GDF15 is required for the development of apoptosis induced by activated mTORC1. A stable C2C12 cell line that overexpresses TSC1 shRNA was transfected with control and GDF15 siRNAs (each at 20 nM, Santa Cruz Biotechnology) to silence GDF15. Flow cytometry was performed 4 days after transfection. Activation of mTORC1 induces apoptotic markers (top panel), but the induced apoptosis was significantly suppressed by transfected GDF15 siRNA (lower panel). *n* = 3, *p < 0.05. (h) GDF15 increases the levels of autophagic marker, LC3, revealed by Western blot analysis. Increased protein levels of both LC3‐I and LC3‐II, 3 days after transfection of GDF15 into C2C12 cells. (i) GDF15 induces protein oxidation (DNP), polyubiquitination (Ubi), and activation of Smad3 and FoxO1. C2C12 myotubes were treated with recombinant GDF15 protein at 1 and 10 ng/ml for 48 hr. Western blot analysis is shown. Note that GDF15 phosphorylates Smad3 and dephosphorylates FoxO1. (j) GDF15 increases the activity of FoxO's and Smad's reporter gene. GDF15‐expressing plasmid was cotransfected into C2C12 cells, together with a FoxO1 reporter gene 6DBE, or a Smad3 reporter gene 3TP‐Lux, as well as a nonreporter control pCS2‐beta‐Gal, respectively. Luciferase (reporter) and beta‐Gal (nonreporter control) activities were measured 3 days later, and luciferase activity was normalized to beta‐Gal activity

After the identification of GDFs as downstream targets of mTORC1 activation, we examined if they might subserve the mTORC1‐induced oxidative stress and apoptosis in skeletal muscle. Since GDF15 is the most robustly induced gene in the GDF family in mTORC1‐activated muscle, we used GDF15 to perform the further tests. We transfected C2C12 cells with control GFP and GDF15‐GFP plasmids, respectively. One day later, the GFP+ cells were sorted by FACS and then stained with Annexin V (AV) and Propidium Iodide (PI) to identify apoptosis. After GDF15 transfection, each of the apoptotic markers AV and PI are significantly induced (Figure 6a,b). In culture, the GDF15‐transfected cells are consistently positive for cleaved (active form) caspase 3, shown by immunostaining (Figure 6c). Recombinant GDF15 (10 ng/ml, for 4 days) also induces the levels of cleaved caspase 3 in cultured myotubes, as shown by Western blot analysis (Figure 6d). Similarly, treatment of GDF3 and GDF5 (10 ng/ml, for 4 days) also leads to the activation of caspase 3 activity (Figure 6d). We further confirmed the apoptotic effect induced by GDFs in vivo by electroporation of GDF15 plasmid (GDF15‐GFP) and control (GFP) into TA muscle. Fourteen days after GDF15 electroporation, ~9% of the GDF15‐transfected muscle fibers show positive staining for cleaved caspase 3 (Figure 6e,f), but none of the control fibers show positive staining of caspase 3.

We next tested if GDF15 participates in the induction of mTORC1‐induced apoptosis. GDF15 siRNA was transfected into an mTORC1‐activated C2C12 stable cell line harboring TSC1 shRNA. Three days later, the cells were stained with Annexin V and PI. Successful knockdown of GDF15 expression was confirmed by quantitative PCR (Supporting Information Figure S5). As in the case of in vivo mTORC1 activation in skeletal muscle, mTORC1 activation following TSC1 silencing in cultured cells also increases the levels of both apoptotic markers. Silencing GDF15 with GDF15 siRNA significantly reduces the levels of these markers, indicating the GDF15 likely plays a role in the development of apoptosis induced by mTORC1 (Figure 6g). In addition to the activation of caspase‐mediated apoptotic changes, GDF15 also upregulates the autophagic marker LC3 (Figure 6h) and induces protein ubiquitination and oxidation, as demonstrated by the upregulation of dinitrophenyl (DNP) and protein polyubiquitination (ubi; Figure 6i). This suggests that GDF15 mediates mTORC1‐induced degenerative effects by activating several catabolic pathways.

We also investigated the downstream signaling regulated by GDF15 by analyzing recombinant GDF15‐treated myotubes. This allowed us to identify Smad3 and FoxO1 as downstream targets of GDF15. We found that GDF15 treatment induces the phosphorylation of Smad3, but not Smad2, and dephosphorylates FoxO1 (Figure 6i), leading to their activation. GDF‐dependent regulation of Smad3 and FoxO1 was further confirmed by overexpression of GDF15 through transfecting a GDF15‐expressing plasmid in cultured cells (Supporting Information Figure S7). Consistently, GDF15 upregulates the reporter activities of both FoxO1 and Smad3, as demonstrated by cotransfection of a GDF15‐expressing plasmid with the FoxO1 reporter gene 6DBE, or the Smad3 reporter gene 3TP‐Lux, respectively (Figure 6j). Interestingly, the phosphorylation levels of Akt protein appear not to be affected by GDF15 (Figure 6i), indicating that GDF15 only mediates part of mTORC1's effect, with the reduction of Akt phosphorylation/activity in mTORC1‐activated muscle driven by other mechanisms, for example, the S6 kinase/Grb10‐IRS feedback route (Saxton & Sabatini, 2017).

### 2.7 mTORC1 upregulates the expression of GDF15 by phosphorylating STAT3

To determine the mechanism by which mTORC1 increases the expression of GDF15, we performed bioinformatic analysis of GDF15's promoter sequence. We found several consensus DNA elements that the transcription factor STAT3 may bind within the promoter. It is known that STAT3 is regulated by mTORC1's kinase activity through directly phosphorylating the STAT3 protein at serine 727 (S727), and this phosphorylation increases STAT3's activity (Laplante & Sabatini, 2013; Wen, Zhong, & Darnell, 1995; Yokogami, Wakisaka, Avruch, & Reeves, 2000). Therefore, we speculated that mTORC1 transcriptionally upregulates GDF15 by activating the transcription factor STAT3.

**Figure 7:**
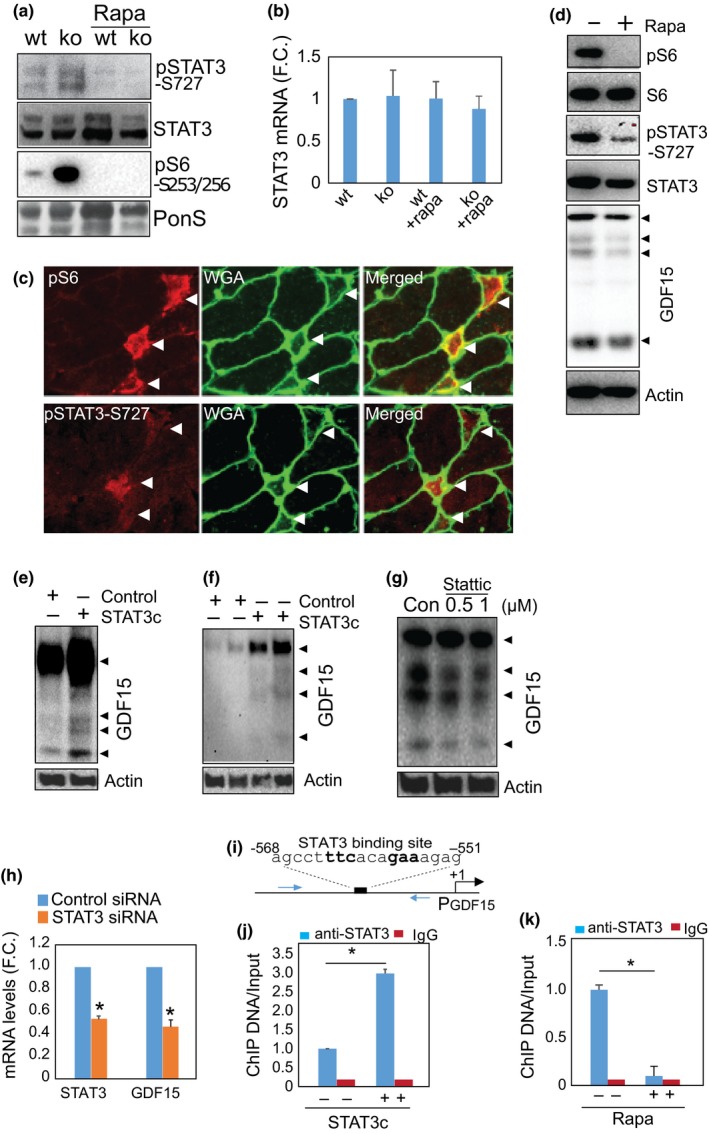
mTORC1‐activated STAT3 transcriptionally upregulates GDF15. (a) mTORC1 activity regulates the phosphorylation of STAT3 in mTORC1‐activated muscle. Western blot analysis was performed with TA muscle lysate. *n* = 3, representative picture was shown. Note, mTORC1‐induced STAT3 phosphorylation is suppressed by rapamycin treatment. (b) mTORC1 activity does not regulate STAT3 mRNA levels. Quantitative PCR was performed to measure mRNA levels in gastrocnemius muscle. *n* = 4 per group. (c) STAT3 is phosphorylated in pS6+ muscle fibers in aged muscle. Immunostaining was performed to detect phosphorylated S6 and STAT3 (S727) with specific antibodies. Arrowheads indicate positively stained muscle fibers. (d) Inhibition of mTORC1 activity suppresses STAT3 phosphorylation and the expression of GDF15 in cultured myotubes. Rapamycin, 2 nM, for 24 hr. Experiments were repeated three times, and representative images were shown. (e and f) Overexpression of STAT3 increases the expression of GDF15 in vitro and in vivo. Constitutive active STAT (STAT3c) was transfected into C2C12 cells (e) or electroporated into TA muscle (f). GDF15 levels were examined by Western blot analysis. (g) Inhibition of STAT3 activity suppresses the expression of GDF15. STAT3 inhibitor, Stattic, was used to treat cultured myotubes for 24 hr, and GDF15 levels were examined by Western blot analysis. Experiments were repeated three times and representative image was shown. (h) Silencing STAT3 reduces GDF15 expression. siRNA against STAT3 was transfected into C2C12 cells and RNA was harvested 4 days later for quantitative PCR. *n* = 4, *p < 0.05. (i, j, and k) STAT3 binds to GDF15 promoter. Consensus sequence of STAT3 binding is present in the promoter sequence of GDF15 (i). ChIP assay was performed with STAT3 transfected C2C12 cells (j) and rapamycin‐treated C2C12 myotubes (k). *n* = 4 each, *p < 0.05. Increased STAT3 level increases STAT3's DNA binding, whereas rapamycin treatment reduces STAT3 binding to the GDF promoter

We first examined if STAT3‐S727 is phosphorylated at S727 upon the activation of mTORC1 in skeletal muscle. Shown in Figure 7a with a specific antibody against S727 of STAT3 protein, the phosphorylation level of STAT3 at serine 727 is indeed elevated, and rapamycin treatment reduces the level of phosphorylation. This mTORC1‐dependent regulation of STAT3 is mainly at the posttranslational level, since mRNA levels of STAT3 remain unchanged (Figure 7b). Consistently, the aged muscle fibers with activated mTORC1 also show the positive staining of pSTAT3‐S727 (Figure 7c). We also confirmed that in cultured C2C12 myotubes, inhibition of mTORC1 activity with rapamycin suppresses STAT3‐S727's phosphorylation and GDF15 expression (Figure 7d). Increasing STAT3 activity by overexpression of a constitutively active STAT3 (STAT3c) is able to induce GDF15 expression in both cultured cells (Figure 7e) and mouse skeletal muscle (Figure 7f). On the other hand, inhibition of STAT3 activity with a small‐molecule inhibitor, Stattic, suppresses GDF15 expression (Figure 7g and Supporting Information Figure S8). Decreasing STAT3 expression with STAT3 siRNA also reduces GDF15 level (Figure 7h).

To further examine whether STAT3 transcriptionally upregulates GDF15 via directly binding to the GDF15 promoter sequence, we performed chromatin immunoprecipitation (CHIP) assay. A consensus DNA element that can bind with STAT3, agcct**ttc**aca**gaa**agag, is located at −551 to −568, upstream of the transcription initiation site of GDF15. We demonstrated that overexpression of STAT3 increases STAT3's binding to this DNA sequence, whereas rapamycin treatment reduces STAT3's binding to the consensus sequence in the GDF15 promoter in cultured myotubes (Figure 7i–k).

### 2.8 Inhibition of mTORC1 with rapamycin blocks GDF expression and oxidative stress in elderly mice and prevents age‐related muscle fiber loss

**Figure 8:**
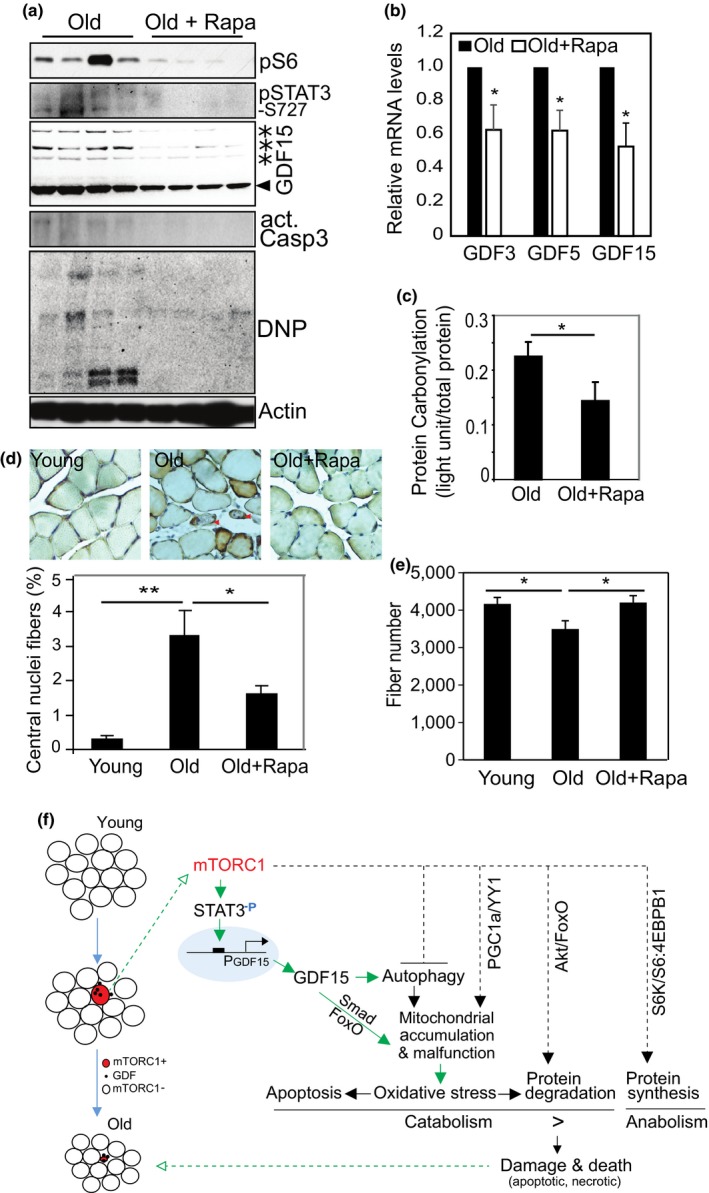
Inhibition of mTORC1 activity in aged animals inhibits GDF15 expression and prevents age‐related oxidative stress and fiber loss. (a) Rapamycin treatment (14 ppm) for ~830 days beginning at age ~270 days suppresses muscle mTORC1 pathway activity, STAT3 phosphorylation, protein oxidation (DNP), cleaved caspase 3, and the protein expression of GDF15. Western blot analysis was performed with specific antibodies. (b) The mRNA expression levels of GDF3, 5, and 15 are all reduced after rapamycin treatment, measured by quantitative PCR. *n* = 4, *p < 0.05. (c) Rapamycin treatment reduces aging‐induced protein carbonylation. Total muscle proteins were extracted from gastrocnemius muscle. The value for protein carbonylation is measured by ELISA and normalized to the total protein input. *n* = 4, *p < 0.05. (d) Rapamycin prevents aging‐induced central myonuclear localization. HE staining was performed and centrally located nuclei were counted in TA muscle. Representative images were shown, and abnormal fibers with centrally localized nuclei were indicated with arrow head. Results are percentage of cells with central nuclei over total fiber count. 200 fibers counted per sample, *n* = 4 muscles, *p < 0.05, **p < 0.01. (e) Age‐dependent loss of muscle fiber number is prevented by rapamycin treatment. Total number of muscle fibers in TA muscle was counted. Young: *n* = 4; old: *n* = 10; old with rapamycin: *n* = 10, *p < 0.05. (f) Schematic diagram to show the hypothetical mechanism underlying mTORC1‐GDF‐dependent muscle fiber loss during aging. Note, the signaling cascade linked by the green arrows was from in the current study, whereas the black dash line arrows from previous reports

Finally, we examined if inhibiting mTORC1 activation in aged skeletal muscle could prevent age‐related deleterious changes in the muscle. For this experiment, mice were treated with rapamycin (14 ppm) from ~270 days to ~900–1,099 days of age, as described (Harrison et al., 2009; Miller et al., 2014). Age‐dependent induction of GDFs and phosphorylated STAT3 (S727) was observed in both human and mouse muscle (Supporting Information Figure S9). Rapamycin treatment in this setting efficiently inhibits the phosphorylation of S6 and STAT3 protein, and simultaneously reduces the expression of GDF15, protein oxidation (carbonylation, DNP), and cleaved caspase 3 (Figure 8a). A similar result is observed at the transcriptional levels for GDF3, GDF5, and GDF15 (Figure 8b). We also evaluated the level of intracellular oxidative stress by measuring oxidized proteins and found that age‐induced oxidative stress is suppressed by rapamycin treatment (Figure 8c). The centrally located nuclei that are associated with muscle damage and regeneration and are induced in aged muscle are significantly fewer in rapamycin‐treated than control, aged animals (Figure 8d). Age‐dependent reduction in muscle fiber number is also recovered by rapamycin treatment. The fiber number in the rapamycin‐treated muscle is significantly higher than in the age‐matched control group (Figure 8e).

## 3 DISCUSSION

Understanding the regulatory mechanisms underlying muscle aging is critical to the development of therapeutic interventions against sarcopenia. We report here that mTORC1 signaling is dramatically activated in a subset of aging muscle fibers and that these same fibers show degenerative changes. Inhibition of mTORC1 activity reduces oxidative stress and muscle fiber damage, and it prevents fiber damage and loss in aged muscle. Therefore, the level of mTORC1 activity in muscle fibers is closely associated with phenotypic changes that occur in naturally aging muscle.

To understand how activated mTORC1 leads to muscle damage and loss, we examined a TSC1‐null mouse model (Bentzinger et al., 2013; Castets et al., 2013; Tang et al., 2014). We show that chronic activation of mTORC1 induces a degenerative muscle phenotype consisting of abnormal and dysfunctional mitochondria, oxidative stress, apoptosis, and fiber death. These mTORC1‐driven changes are remarkably similar to those observed in normal, aged muscle (Arking, 2006; Demontis et al., 2013; Hepple, Ross, & Rempfer, 2004; Larsson et al., 1978; Marzetti et al., 2009; Miller et al., 2008; Murakoshi et al., 1985; Narici & Maffulli, 2010; Tandler & Hoppel, 1986). Further, we show mTORC1‐induced mitochondrial oxidative stress and catabolism is linked to the mTORC1‐dependent activation of novel STAT3‐GDF signaling, in addition to the previously known FoxO signaling pathway.

It has been previously reported that sustained activation of mTORC1 inhibits autophagy and leads to late‐onset myopathy in a TSC1 null mouse model (Castets et al., 2013). However, the mechanisms operant here appear to be complex and cannot be explained by impaired autophagy alone. Based on the evidence we present here of activated FoxO‐ and GDFs‐mediated catabolic pathways, as well as the well‐known anabolic function of mTORC1 (protein synthesis and mitochondrial biogenesis; Saxton & Sabatini, 2017), we propose that the myopathy seen in mTORC1‐hyperactive muscle results from a complex process of anabolism and catabolism—their imbalance ultimately leading to muscle fiber damage and loss (Figure 8f). mTORC1 can directly activate FoxO‐ and GDF‐mediated catabolic signaling, independent of autophagy. mTORC1 directly phosphorylates STAT3 at the serine 727 site (Laplante & Sabatini, 2013; Wen et al., 1995; Yokogami et al., 2000). We advance this observation by demonstrating that activated STAT3 regulates GDF15 at the transcriptional level through binding to the cis DNA element in the GDF15 promoter sequence. Therefore, STAT3 is a novel mediator situated between mTORC1's kinase activity and GDF15 gene expression. These findings link mTORC1 to the GDF family, and they add an additional catabolic signaling pathway, beyond the previously known S6K‐Akt‐FoxO feedback pathway, that mediates the degenerative effect of mTORC1 (Figure 8f).

Interestingly, we show here also that GDF15 itself facilitates autophagy by upregulating LC3. This seemingly contradictory regulation may represent a cellular effort to maintain homeostasis in mTORC1‐activated muscle. Further, abnormal mitochondria accumulation seen in the mTORC1‐activated muscles would seem unlikely to result solely from the inhibition of autophagy/passive accumulation. Active mitochondrial proliferation induced by mTORC1 is a separate, important contributor to the observed mitochondrial accumulation (Cunningham et al., 2007; Koyanagi et al., 2011; Morita et al., 2013) by promoting translation of mitochondria‐related mRNAs via inhibition of the eukaryotic translation initiation factor 4E (eIF4E)‐binding proteins (4E‐BPs; Morita et al., 2013).

It is worth noting that although mTORC1 is hyperactive in all muscle fibers in the TSC1 ko mice, apoptotic changes were only sporadically seen by immunostaining for cleaved caspase 3. This was likely due to variation between cells in the intracellular balance between oxidative stress and self‐defense/antioxidant systems from cell to cell. The negatively stained fibers may well become positive days/weeks later. Consistent with this hypothesis, the “few apoptotic fibers” seen at 9 months old (Figure 3e) progresses to nearly 50% fiber loss at the 18 months (Figure 2g). The accumulated damage over time is much more than the “few apoptotic fibers” seen early on. In addition, oxidative stress induced by hyperactive mTOR also leads to protein degradation, autophagy, and necrotic changes (Figure 8f). In these cases, the fiber damage and death is not through the apoptotic pathway. In future studies, it might be possible to use laser microdissection to isolate out the pS6+ fibers from aging muscle—this would allow us to study mTORC1‐related events in individual aging fiber in greater detail.

mTORC1‐induced muscle fiber damage and loss appears to be a progressive process occurring over time. In TSC1 ko mice, the elevated mTORC1 activity does not cause a severe muscle degenerative phenotype until late adulthood, despite the fact that mTORC1 has been activated since embryonic development. We show also that oxidative stress and apoptosis‐related genes are significantly induced only at an older age. Similarly, in normal aging mice, activation of mTORC1 in muscle fibers can be detected at middle age (12 months old, Supporting Information Figure S1), but we saw no significant degenerative morphology until 30 months of age. We theorize that mTORC1‐induced mitochondrial enlargement/dysfunction progresses until some turning point at which mitochondrial free‐radical generation (MOS) overwhelms resident antioxidant systems, thereby causing catabolism. This framework implies that early preventive treatment via inhibition of mTORC1 signaling may in part prevent sarcopenia.

We tested this idea by treating mice with rapamycin (14 ppm) from the age of 270 days to the age of 900–1,099 days. The rapamycin treatment did suppress mTORC1 activity, GDF15 expression, and apoptosis, and prevent fiber loss (Figure 8). Additionally, hyperactive mTORC1 increased the level of type I myosin heavy chain, but decreased type IIb, in TSC1 ko mice (Supporting Information Figure S10a). Inhibition of mTORC1 activity with rapamycin in aged muscle led to reduction of MHC I (Supporting Information Figure S10b). Therefore, mTORC1 activity may also contribute to the age‐dependent conversion into slow oxidative fibers.

However, the protocol of rapamycin treatment that we used in this study did not significantly rescue the age‐related decrease in muscle fiber size and wet weight, although there may be a trend in this direction (Supporting Information Figure S11). Although it is reasonable to speculate that this could be due to rapamycin inhibiting the regenerative effort in aged muscle, the expression of embryonic myosin heavy chain (MyH3), a molecular marker of muscle regeneration, was not altered significantly by rapamycin treatment (Supporting Information Figure S12), indicating that rapamycin treatment, at least at this dosage, did not increase or decrease muscle regenerative capacity. We suspect that long‐term, continuous treatment with rapamycin, as we used in this study, reduced the essential intracellular anabolic activity that is maintained by a basal level of mTORC1 in normal, healthy fibers during aging. This effect would compromise any potential beneficial effect of rapamycin. It is possible that a different titration of the dose of rapamycin and/or an optimized treatment protocol (e.g., intermittent rather than continuous duration, shorter duration of treatment) might result in a net muscle‐protective effect.

Growth differentiation factors are known to be associated with many diseases states. For example, GDF15 plays a role in cancer cachexia (Lerner et al., 2015; Weide et al., 2016), mitochondrial diseases (Kalko et al., 2014; Montero et al., 2016; Yatsuga et al., 2015), and as a mediator of intensive care unit (ICU)‐acquired muscle weakness (Bloch et al., 2015). Its expression has even been associated with mortality (Wiklund et al., 2010). In addition, GDF15 regulates appetite and is related to weight loss and cancer‐related anorexia (Breit et al., 2011; Johnen et al., 2007; Lerner et al., 2015; Tsai et al., 2016, 2013, 2014; Villars et al., 2017), likely functioning through the receptors of the previously reported TGF‐β receptor II (Johnen et al., 2007) and the recently discovered GFRAL (GDNF Family Receptor Alpha Like; Emmerson et al., 2017; Hsu et al., 2017; Mullican et al., 2017; Yang et al., 2017) in the central nervous system. Anorexia could, of course, be a contributor to the loss of body weight in the mTORC1‐activated mice we studied. However, it is unlikely that the mTORC1‐induced muscle damage and loss is entirely due to GDF15‐related anorexia because (a) mTORC1‐activated muscle reveals a heterogeneous phenotype of muscle fibers with the presence of both the proliferated/enlarged and the degenerated/shrunken muscle fibers; (b) loss of the skeletal muscle is more prominent than the loss of body weight; (c) GDFs can directly induce catabolism in muscle cells in vitro and in vivo; (d) mTORC1 also activates FoxO‐mediated catabolic pathways through inhibition of Akt, as previously reported, independent of GDFs. On the other hand, GDF15 has also been reported also to have beneficial effects as an anti‐inflammatory and in a protective role in cardiac muscle (Kempf et al., 2006, 2011). High levels of circulating GDF15 in pregnant women (Moore et al., 2000) appear harmful to neither their muscle nor overall health. It is thus likely that the ultimate effect of GDFs is context‐dependent.

The catabolic effect of GDF15 in skeletal muscle that we propose may act through GFRAL receptor as it does in the central nervous system, since GFRAL is also expressed in cultured myotubes and skeletal muscle (Supporting Information Figure S13). It is also possible that GDF3, 5, and 15 regulate muscle catabolism through an unknown intracellular mechanism, other than the ligand‐receptor interaction, which remains to be elucidated. Downstream effectors of GDFs‐induced oxidative stress and catabolism may include Smad3 and FoxO1, since these have been reported to compromise mitochondrial function and induce apoptosis (Black et al., 2007; Jang et al., 2002; Michaeloudes, Sukkar, Khorasani, Bhavsar, & Chung, 2011; Wildey, Patil, & Howe, 2003). FoxO is also able to induce Bim gene expression and apoptosis (McLoughlin et al., 2009; Shukla, Rizvi, Raisuddin, & Kakkar, 2014). Our finding of GDF‐induced dephosphorylation of FoxO uncovers a novel posttranslational activation of FoxO, independent of Akt activity (Figure 6i).

Catabolism induced by elevated GDFs, MOS, and activated FoxO may lead directly to muscle fiber loss. It has been reported previously that dysfunctional mitochondria and apoptosis occur in aging skeletal muscle (Bratic & Larsson, 2013; Bua, McKiernan, Wanagat, McKenzie, & Aiken, 2002; Chabi et al., 2008; Cheema et al., 2015; Demontis et al., 2013; Dirks & Leeuwenburgh, 2005; Herbst, Johnson, Hynes, McKenzie, & Aiken, 2013; Hiona et al., 2010; Jang et al., 2010; McArdle et al., 1999; Pistilli, Siu, & Alway, 2006; Short et al., 2005; Stephen, Michael, & Parco, 2011; Whitman, Wacker, Richmond, & Godard, 2005), resulting in apoptotic and necrotic fiber loss (Cheema et al., 2015). Apoptotic muscle fiber death appears to be different from that in mononuclear cells because decay of an individual nucleus is insufficient to cause death of multinucleated muscle fibers. However, progressive, accumulated nuclear and cellular apoptotic changes may lead to gradual, perhaps segmental muscle fiber death over a long period of time (e.g., aging; Cheema et al., 2015; Dupont‐Versteegden, 2005). GDFs and MOS increase free radicals, resulting in oxidation and ubiquitination of cellular components, autophagy, and thereby ultimately apoptotic and/or necrotic cell death. Since GDF3, 5, and 15 are all induced by mTORC1 and since they functionally overlap, a muscle‐specific, triple knockout of GDF3, 5, and 15 might help define GDFs' unique role in mTORC1‐induced muscle degeneration.

Lastly, it deserves mention that our findings are in some ways consistent with the “mitochondrial theory of aging” (Beckman & Ames, 1998; Harman, 1972; Miquel, Economos, Fleming, & Johnson, 1980). Our mTORC1‐centric view advances this theory by identifying mTORC1‐GDF as a specific inducer of MOS in the aging of one particular tissue—skeletal muscle. Precisely how mTORC1 is activated in a subset of muscle fibers during aging remains unknown, but loss or damage of motor neuron during aging may contribute to mTORC1's activation, since denervation indeed activates mTORC1 (Tang et al., 2014). IGF‐1, a conventional upstream regulator of mTORC1, however, does not regulate the serine 727 phosphorylation and GDF15 expression in cultured myotubes, indicating that GDF15 expression is regulated by IGF/Akt‐independent activation of mTORC1 (Supporting Information Figure S14).

In summary, we have discovered a novel molecular mechanism underlying age‐related muscle fiber damage and loss, linking mTORC1 with GDFs, and we suggest that mTORC1‐GDF is a contributing pathway to the development of sarcopenia. Cell damage and death occurring via this mTORC1‐GDF pathway may also occur in other tissues during aging, since these genes are widely expressed.

## 4 MATERIALS AND METHODS

### 4.1 Human latissimus dorsi and mouse muscle samples

Human latissimus dorsi muscles were taken during thoracic surgery with approval from the Stanford Institutional Review Board. Patients fasted for approximately 9 hr prior to the surgery and taking of biopsies. We excluded patients with more than mild pulmonary dysfunction, cancer beyond stage I, or use of steroids of other drugs that can affect muscle physiology. Age and sex are shown in Supporting Information Figure S7.

The animal care and experimental procedures followed a protocol approved by the University of Michigan Committee on Use and Care of Animals and the University of California San Diego Institutional Animal Care and Use Committee. The generation of TSC1lox/lox mice with exons 17 and 18 of Tsc1 flanked by loxP sites by homologous recombination was done as described previously (Tang et al., 2014). Mice were fed with regular chow and water ad libitum. TSC1 ko and control animals were injected with rapamycin intraperitoneally (6 mg/kg body weight, every other day) for 8 weeks, beginning at the 5 weeks of age. Rapamycin (LC Laboratories; Woburn, MA) was dissolved in 100% ethanol, stored at −20°C, and further diluted in an aqueous solution of 5.2% Tween 80 and 5.2% PEG 400 (final ethanol concentration, 2%) immediately before use. Hindlimb muscles were collected for sample analysis. Fresh muscle tissues from female C57BL/6 mice were also used to examine the expression levels of protein and RNA between young and old.

HET3 female mice were treated with rapamycin as described (Harrison et al., 2009; Miller et al., 2014). Treatment of rapamycin (14 ppm) started at 270 days of age and evaluated at ~900–1,096 days of age. Untreated, age‐matched, or young (2 months) mice were used as controls. Formalin‐fixed muscle samples (UM‐HET3) were used for histological study. Fresh muscle samples (UT‐HET3) were used for protein and RNA analysis.

### 4.2 Transcriptomic profiling of gene expression and data analysis

Total RNA was harvested from gastrocnemius muscle and subjected to Agilent single color gene microarray, performed by the Human Immune Monitoring Center at Stanford University. Data were normalized and analyzed using the GeneSpring GX and DAVID bioinformatics tools (http://david.ncifcrf.gov/content.jsp?file=citation.htm).

### 4.3 Gene transfection and electroporation

GDF15‐GFP was constructed by PCR amplification of the cDNA of human GDF15 and ligation into the pCMV6‐AC‐GFP vector between Sgf1 and Mlu1. The plasmid was transfected into cultured cells with lipofectamine 2000 (Invitrogen). Electroporation of plasmid into tibialis anterior (TA) muscle was performed as previously described^17^. Briefly, two hours prior to electroporation, TA muscles were injected with 50 μl of hyaluronidase (0.5 μ/μl, Sigma‐Aldrich) to increase the transfection efficiency. ECM830 electroporator (BTX) was used to deliver eight pulses of 75 v/cm, 20‐ms duration, and 100‐ms interval. Twenty micrograms of plasmid (GDF15‐GFP or control GFP) were electroporated into TA muscles, and muscle samples were harvested 14 days later and fixed in 4% paraformaldehyde for immunostaining.

C2C12 cells were stably transfected with TSC1 shRNA (open biosystems, plko1 TSC1#217), and activated mTORC1 activity was confirmed by elevated pS6 levels. GDF15 siRNA was purchased from Santa Cruz Biotechnology. Twenty nM of control siRNA or GDF15 siRNA was transfected to TSC1 shRNA stable cell lines for 4 days, followed by AV and PI staining, and flow cytometry.

### 4.4 Flow cytometry

Flowcytometric analysis was performed by standard methods with an LSR II flow cytometer unit (BD biosciences). Cells were digested and immunostaining was performed with Annexin V and PI apoptosis detection kits (BD Biosciences). After staining, the cells were filtered through a cell strainer and 100,000–1,000,000 events were acquired for each sample. Flow cytometry data were analyzed with BD FACSDiva software.

### 4.5 Western blotting analysis and real‐time PCR

Recombinant GDF3, 5, and 15 were purchased from Biovision. Protein expression and protein phosphorylation were detected by Western blotting analysis following standard procedures. Briefly, ten micrograms of total protein extracted from muscles was loaded onto 4%–12% SDS‐PAGE gels and transferred to nitrocellulose membranes for antibody detection. Antibodies against mouse and human GDF15 were purchased from Santa Cruz Biotechnology, Inc. The remainder of the antibodies was purchased from Cell Signaling Technology. Antibodies were used at 1:1,000 dilutions for Western blot analysis. Phosphorylation sites that the antibodies can recognize are: pmTOR (S2448), pS6K1 (T389), pS6 (S235/236), pFoxO1/3 (T24/T32), pFoxO1 (S256), pSmad2 (S465/467), pSmad3 (S423/S4250, and pSTAT3 (S727).

Gene expression levels were detected by real‐time PCR. RNA was reverse‐transcribed with oligo (dT) primer and SuperScript II (Invitrogen), and 1/20 of the cDNA mixture served as template for the PCR reactions. Real‐time PCR was performed on an iCycler (Bio‐Rad, Hercules, CA) by using SYBR Green SuperMix (Bio‐Rad).

### 4.6 Chromatin immunoprecipitation

Cultured C2C12 cells were transfected with STAT3‐expressing plasmid, or differentiated into myotubes and treated with rapamycin for 24 hr. Cells were then fixed with formaldehyde (1%) for 10 min, followed by 125 mM glycine for 5 min. Cells were then washed, lysed, and centrifuged at 950 *g* for 5 min to collect the nuclei. Nuclear lysate was sonicated to break down chromatin (~500 bp) and incubated with Protein A Agarose/salmon sperm DNA. After centrifugation, the supernatant (input DNA, an aliquot saved for measuring input DNA amount) was incubated with STAT3 antibody and IgG (negative control), respectively, overnight at 4°C, and then added with Protein A Agarose for another 2 hr. This mixture was centrifuged at 220 *g* to precipitate the protein–chromatin complex. After washing, the protein–chromatin complex was eluted from Protein A Agarose, and de‐crosslinked with NaCl (0.2 M) at 65°C overnight. The protein–chromatin mixture was treated with proteinase K (0.2 mg/ml) at 60°C for 1 hr. The resulting mixture was further extracted with phenol/chloroform for PCR amplification. Quantitative PCR was performed with primers (Forward: 5′‐AAGGTCACATGGGACCGCGG; Reverse: 5′‐TGCCCTGGGCGAGCTGCTGA). The amount of input DNA was measured by PCR with beta‐actin primers (Forward: 5′‐AGGCGGACTGTTACTGAGCTG; Reverse: 5′‐CAACCAACTGCTGTCGCCTT) as normalization control.

### 4.7 Muscle morphology, histology, immunostaining, SDH staining, DHE staining, and cross‐sectional area (CSA)

Muscle samples were either embedded in paraffin (for HE staining) or freshly frozen (for SDH staining). Paraffin embedded samples were sectioned at 5 μm thickness for standard HE staining. Frozen muscle tissues (12 μm) were sectioned on a Leica cryostat. Fresh sections without fixation were used for SDH staining, while fresh sections fixed in 2% paraformaldehyde for 15 min were used for immunostaining. Antibodies against activated/cleaved caspase 3 and pS6 were purchased from Cell Signaling and used at 1:100 dilutions. For negative control, normal serum was used to replace primary antibodies. Secondary antibodies (either anti‐rabbit, or anti‐mouse) conjugated with Cy5 or FITC were used at 1:500 dilution.

Succinate dehydrogenase (SDH) staining was performed by incubating fresh muscle tissue sections in 0.1 M phosphate buffer, pH 7.6, 5 mM EDTA, pH 8.0, 1 mM KCN, 21.8 mg/ml sodium succinate, and 1.24 mg/ml nitroblue tetrazolium for 20 min at room temperature. Sections were then rinsed, dehydrated, and mounted before microscopic visualization. Sections were observed using an Axiophot microscope (Carl Zeiss, Thornwood, NY) equipped with fluorescence optics.

Zeiss LSM710 Laser Scanning microscope and Zen software system (Carl Zeiss) were used to take confocal images.

Dihydroethidium (DHE) staining was performed on snap‐frozen muscle samples. DHE was purchased from Invitrogen and reconstituted in anhydrous DMSO (Sigma‐Aldrich) at a stock concentration at 10 mM. The staining solution was prepared fresh before use by 1–1,000 dilution of the stock DHE solution with 1XPBS. The DHE/PBS solution was placed over cryosections (20 μm) and incubated for 10 min in a dark chamber. The reaction was stopped by washing in 1XPBS three times. Slides were mounted in Prolong Gold antifading reagent (Invitrogen) and imaged by fluorescent microscopy (Leica). The cross‐sectional area (CSA) was measured with ImageJ software after taking the SDH‐stained pictures. No less than 500 fibers were counted per specimen.

Total muscle fibers in TA muscle in young, old, and old rapamycin‐treated samples were counted. Cross sections of the same location of each muscle were created and stained with WGA. Fiber number was counted using ImageJ software. In TSC1 ko muscle, we counted fibers specifically either from the lateral head of the gastrocnemius muscle or TA muscle: The muscle was sectioned and stained with WGA, and a 10X image was then taken from the same region, of the muscle. All fibers in this one, low‐power field from this region were then counted. Results are presented as gross fiber counts.

One‐way analysis of variance (ANOVA) was used to determine significance when there were more than three groups for comparison, followed by Tukey post hoc test. Student's *t*‐test was used to evaluate the statistical significance between two groups. *p* < 0.05 was considered as statistically significant.

### 4.8 Transmission electron microscopy

Mice were anesthetized by intraperitoneal injection of ketamine (100 mg/kg) and xylazine (10 mg/kg) and then perfused via cardiac puncture with 4% paraformaldehyde (PFA) in 0.1 M cacodylate buffer, pH 7.4. Soleus muscles from the wild‐type and the TSC^−/−^ mice were isolated and immersed in the same fixative for 24 hr at 4°C. Muscles were then fixed in 1.5% glutaraldehyde, 3% PFA, 5% sucrose in 0.1 M cacodylate buffer, pH 7.4, at room temperature for 2 hr. The samples were postfixed in 1% OsO4 in the same buffer for 1 hr, stained *en bloc* in 2% uranyl acetate in 10% ethanol for 1 hr, dehydrated in ethanol, and embedded in LX112. Tissue sections were stained with uranyl acetate and lead citrate and examined in a Jeol JEM 1200EX II electron microscope (JEOL USA, Peabody, MA). Magnification is indicated on each image.

## CONFLICT OF INTEREST

None declared.

## References

[acel12943-bib-0001] Arking , R. (2006). Biology of aging: Observations and principles. (3 ed.). New york, NY: Oxford University Press.

[acel12943-bib-0002] Baar, E. L. , Carbajal, K. A. , Ong, I. M. , & Lamming, D. W. (2016). Sex‐ and tissue‐specific changes in mTOR signaling with age in C57BL/6J mice. Aging Cell, 15(1), 155–166. 10.1111/acel.12425 26695882 PMC4717274

[acel12943-bib-0003] Beckman, K. B. , & Ames, B. N. (1998). The free radical theory of aging matures. Physiological Reviews, 78(2), 547–581. 10.1152/physrev.1998.78.2.547 9562038

[acel12943-bib-0004] Bentzinger, C. , Lin, S. , Romanino, K. , Castets, P. , Guridi, M. , Summermatter, S. , … Rüegg, M. A. (2013). Differential response of skeletal muscles to mTORC1 signaling during atrophy and hypertrophy. Skeletal Muscle, 3(1), 6 10.1186/2044-5040-3-6 23497627 PMC3622636

[acel12943-bib-0005] Bentzinger, C. F. , Romanino, K. , Cloëtta, D. , Lin, S. , Mascarenhas, J. B. , Oliveri, F. , … Rüegg, M. A. (2008). Skeletal muscle‐specific ablation of raptor, but not of rictor, causes metabolic changes and results in muscle dystrophy. Cell Metabolism, 8(5), 411–424. 10.1016/j.cmet.2008.10.002 19046572

[acel12943-bib-0006] Black, D. , Lyman, S. , Qian, T. , Lemasters, J. J. , Rippe, R. A. , Nitta, T. , … Behrns, K. E. (2007). Transforming growth factor beta mediates hepatocyte apoptosis through Smad3 generation of reactive oxygen species. Biochimie, 89(12), 1464–1473. 10.1016/j.biochi.2007.09.001 17936489 PMC2151473

[acel12943-bib-0007] Bloch, S. A. , Lee, J. Y. , Syburra, T. , Rosendahl, U. , Griffiths, M. J. , Kemp, P. R. , & Polkey, M. I. (2015). Increased expression of GDF‐15 may mediate ICU‐acquired weakness by down‐regulating muscle microRNAs. Thorax, 70(3), 219–228. 10.1136/thoraxjnl-2014-206225 25516419 PMC4345798

[acel12943-bib-0008] Bodine, S. C. , Stitt, T. N. , Gonzalez, M. , Kline, W. O. , Stover, G. L. , Bauerlein, R. , … Yancopoulos, G. D. (2001). Akt/mTOR pathway is a crucial regulator of skeletal muscle hypertrophy and can prevent muscle atrophy in vivo. Nature Cell Biology, 3(11), 1014–1019. 10.1038/ncb1101-1014 11715023

[acel12943-bib-0009] Bratic, A. , & Larsson, N. G. (2013). The role of mitochondria in aging. Journal of Clinical Investigation, 123(3), 951–957. 10.1172/JCI64125 23454757 PMC3582127

[acel12943-bib-0010] Breit, S. N. , Johnen, H. , Cook, A. D. , Tsai, V. W. W. , Mohammad, M. G. , Kuffner, T. , … Brown, D. A. (2011). The TGF‐β superfamily cytokine, MIC‐1/GDF15: A pleotrophic cytokine with roles in inflammation, cancer and metabolism. Growth Factors, 29(5), 187–195. 10.3109/08977194.2011.607137 21831009

[acel12943-bib-0011] Bua, E. A. , McKiernan, S. H. , Wanagat, J. , McKenzie, D. , & Aiken, J. , M. (2002). Mitochondrial abnormalities are more frequent in muscles undergoing sarcopenia. Journal of Applied Physiology (1985), 92(6), 2617–2624. 10.1152/japplphysiol.01102.2001 12015381

[acel12943-bib-0012] Buxton, P. , Edwards, C. , Archer, C. W. , & Francis‐West, P. (2001). Growth/differentiation factor‐5 (GDF‐5) and skeletal development. The Journal of Bone and Joint Surgery‐American Volume, 83‐A Suppl 1(Pt 1), S23–30. 10.2106/00004623-200100001-00004 11263662

[acel12943-bib-0013] Castets, P. , Lin, S. , Rion, N. , Di Fulvio, S. , Romanino, K. , Guridi, M. , … Rüegg, M. A. (2013). Sustained activation of mTORC1 in skeletal muscle inhibits constitutive and starvation‐induced autophagy and causes a severe, late‐onset myopathy. Cell Metabolism, 17(5), 731–744. 10.1016/j.cmet.2013.03.015 23602450

[acel12943-bib-0014] Chabi, B. , Ljubicic, V. , Menzies, K. J. , Huang, J. H. , Saleem, A. , & Hood, D. A. (2008). Mitochondrial function and apoptotic susceptibility in aging skeletal muscle. Aging Cell, 7(1), 2–12. 10.1111/j.1474-9726.2007.00347.x 18028258

[acel12943-bib-0015] Cheema, N. , Herbst, A. , McKenzie, D. , & Aiken, J. M. (2015). Apoptosis and necrosis mediate skeletal muscle fiber loss in age‐induced mitochondrial enzymatic abnormalities. Aging Cell, 14(6), 1085–1093. 10.1111/acel.12399 26365892 PMC4693455

[acel12943-bib-0016] Chrysovergis, K. , Wang, X. , Kosak, J. , Lee, S.‐H. , Kim, J. S. , Foley, J. F. , … Eling, T. E. (2014). NAG‐1/GDF‐15 prevents obesity by increasing thermogenesis, lipolysis and oxidative metabolism. International Journal of Obesity, 38(12), 1555–1564. 10.1038/ijo.2014.27 24531647 PMC4135041

[acel12943-bib-0017] Cornu, M. , Oppliger, W. , Albert, V. , Robitaille, A. M. , Trapani, F. , Quagliata, L. , … Hall, M. N. (2014). Hepatic mTORC1 controls locomotor activity, body temperature, and lipid metabolism through FGF21. Proceedings of the National Academy of Sciences of the United States of America, 111(32), 11592–11599. 10.1073/pnas.1412047111 25082895 PMC4136616

[acel12943-bib-0018] Cunningham, J. T. , Rodgers, J. T. , Arlow, D. H. , Vazquez, F. , Mootha, V. K. , & Puigserver, P. (2007). mTOR controls mitochondrial oxidative function through a YY1‐PGC‐1alpha transcriptional complex. Nature, 450(7170), 736–740. 10.1038/nature06322 18046414

[acel12943-bib-0019] Demontis, F. , Piccirillo, R. , Goldberg, A. L. , & Perrimon, N. (2013). Mechanisms of skeletal muscle aging: Insights from Drosophila and mammalian models. Disease Models & Mechanisms, 6(6), 1339–1352. 10.1242/dmm.012559 24092876 PMC3820258

[acel12943-bib-0020] Dirks, A. J. , & Leeuwenburgh, C. (2005). The role of apoptosis in age‐related skeletal muscle atrophy. Sports Medicine, 35(6), 473–483. 10.2165/00007256-200535060-00002 15974633

[acel12943-bib-0021] Dupont‐Versteegden, E. E. (2005). Apoptosis in muscle atrophy: Relevance to sarcopenia. Experimental Gerontology, 40(6), 473–481. 10.1016/j.exger.2005.04.003 15935591

[acel12943-bib-0022] Emmerson, P. J. , Wang, F. , Du, Y. , Liu, Q. , Pickard, R. T. , Gonciarz, M. D. , … Wu, X. (2017). The metabolic effects of GDF15 are mediated by the orphan receptor GFRAL. Nature Medicine, 23(10), 1215–1219. 10.1038/nm.4393 28846098

[acel12943-bib-0023] Evans, W. J. , Paolisso, G. , Abbatecola, A. M. , Corsonello, A. , Bustacchini, S. , Strollo, F. , & Lattanzio, F. (2010). Frailty and muscle metabolism dysregulation in the elderly. Biogerontology, 11(5), 527–536. 10.1007/s10522-010-9297-0 20683658

[acel12943-bib-0024] Guridi, M. , Tintignac, L. A. , Lin, S. , Kupr, B. , Castets, P. , & Rüegg, M. A. (2015). Activation of mTORC1 in skeletal muscle regulates whole‐body metabolism through FGF21. Science Signalling, 8(402), ra113 10.1126/scisignal.aab3715 26554817

[acel12943-bib-0025] Harman, D. (1972). The biologic clock: The mitochondria? Journal of the American Geriatrics Society, 20(4), 145–147. 5016631 10.1111/j.1532-5415.1972.tb00787.x

[acel12943-bib-0026] Harrison, D. E. , Strong, R. , Sharp, Z. D. , Nelson, J. F. , Astle, C. M. , Flurkey, K. , … Miller, R. A. (2009). Rapamycin fed late in life extends lifespan in genetically heterogeneous mice. Nature, 460(7253), 392–395. 10.1038/nature08221 19587680 PMC2786175

[acel12943-bib-0027] Hepple, R. T. , Ross, K. D. , & Rempfer, A. B. (2004). Fiber atrophy and hypertrophy in skeletal muscles of late middle‐aged Fischer 344 x Brown Norway F1‐hybrid rats. Journals of Gerontology. Series A, Biological Sciences and Medical Sciences, 59(2), 108–117. 10.1093/gerona/59.2.B108 14999023

[acel12943-bib-0028] Herbst, A. , Johnson, C. J. , Hynes, K. , McKenzie, D. , & Aiken, J. M. (2013). Mitochondrial biogenesis drives a vicious cycle of metabolic insufficiency and mitochondrial DNA deletion mutation accumulation in aged rat skeletal muscle fibers. PLoS ONE, 8(3), e59006 10.1371/journal.pone.0059006 23516592 PMC3596334

[acel12943-bib-0029] Hiona, A. , Sanz, A. , Kujoth, G. C. , Pamplona, R. , Seo, A. Y. , Hofer, T. , … Leeuwenburgh, C. (2010). Mitochondrial DNA mutations induce mitochondrial dysfunction, apoptosis and sarcopenia in skeletal muscle of mitochondrial DNA mutator mice. PLoS ONE, 5(7), e11468 10.1371/journal.pone.0011468 20628647 PMC2898813

[acel12943-bib-0030] Houtkooper, R. H. , Argmann, C. , Houten, S. M. , Cantó, C. , Jeninga, E. H. , Andreux, P. A. , … Auwerx, J. (2011). The metabolic footprint of aging in mice. Scientific Reports, 1, 134 10.1038/srep00134 22355651 PMC3216615

[acel12943-bib-0031] Hsu, J.‐Y. , Crawley, S. , Chen, M. , Ayupova, D. A. , Lindhout, D. A. , Higbee, J. , … Allan, B. B. (2017). Non‐homeostatic body weight regulation through a brainstem‐restricted receptor for GDF15. Nature, 550(7675), 255–259. 10.1038/nature24042 28953886

[acel12943-bib-0032] Inoki, K. , Corradetti, M. N. , & Guan, K. L. (2005). Dysregulation of the TSC‐mTOR pathway in human disease. Nature Genetics, 37(1), 19–24. 10.1038/ng1494 15624019

[acel12943-bib-0033] Jang, C. W. , Chen, C. H. , Chen, C. C. , Chen, J. Y. , Su, Y. H. , & Chen, R. H. (2002). TGF‐beta induces apoptosis through Smad‐mediated expression of DAP‐kinase. Nature Cell Biology, 4(1), 51–58. 10.1038/ncb731 11740493

[acel12943-bib-0034] Jang, Y. C. , Lustgarten, M. S. , Liu, Y. , Muller, F. L. , Bhattacharya, A. , Liang, H. , … Van Remmen, H. (2010). Increased superoxide in vivo accelerates age‐associated muscle atrophy through mitochondrial dysfunction and neuromuscular junction degeneration. The FASEB Journal, 24(5), 1376–1390. 10.1096/fj.09-146308 20040516 PMC2987499

[acel12943-bib-0035] Johnen, H. , Lin, S. , Kuffner, T. , Brown, D. A. , Tsai, V. W. , Bauskin, A. R. , … Breit, S. N. (2007). Tumor‐induced anorexia and weight loss are mediated by the TGF‐beta superfamily cytokine MIC‐1. Nature Medicine, 13(11), 1333–1340. 10.1038/nm1677 17982462

[acel12943-bib-0036] Kalko, S. , Paco, S. , Jou, C. , Rodríguez, M. , Meznaric, M. , Rogac, M. , … Jimenez‐Mallebrera, C. (2014). Transcriptomic profiling of TK2 deficient human skeletal muscle suggests a role for the p53 signalling pathway and identifies growth and differentiation factor‐15 as a potential novel biomarker for mitochondrial myopathies. BMC Genomics, 15, 91 10.1186/1471-2164-15-91 24484525 PMC3937154

[acel12943-bib-0037] Kempf, T. , Eden, M. , Strelau, J. , Naguib, M. , Willenbockel, C. , Tongers, J. , … Wollert, K. C. (2006). The transforming growth factor‐beta superfamily member growth‐differentiation factor‐15 protects the heart from ischemia/reperfusion injury. Circulation Research, 98(3), 351–360. 10.1161/01.RES.0000202805.73038.48 16397141

[acel12943-bib-0038] Kempf, T. , Zarbock, A. , Widera, C. , Butz, S. , Stadtmann, A. , Rossaint, J. , … Wollert, K. C. (2011). GDF‐15 is an inhibitor of leukocyte integrin activation required for survival after myocardial infarction in mice. Nature Medicine, 17(5), 581–588. 10.1038/nm.2354 21516086

[acel12943-bib-0039] Kimball, S. R. , & Jefferson, L. S. (2004). Regulation of global and specific mRNA translation by oral administration of branched‐chain amino acids. Biochemical and Biophysical Research Communications, 313(2), 423–427. 10.1016/j.bbrc.2003.07.014 14684179

[acel12943-bib-0040] Koyanagi, M. , Asahara, S.‐I. , Matsuda, T. , Hashimoto, N. , Shigeyama, Y. , Shibutani, Y. , … Kido, Y. (2011). Ablation of TSC2 enhances insulin secretion by increasing the number of mitochondria through activation of mTORC1. PLoS ONE, 6(8), e23238 10.1371/journal.pone.0023238 21886784 PMC3158755

[acel12943-bib-0041] Laplante, M. , & Sabatini, D. M. (2013). Regulation of mTORC1 and its impact on gene expression at a glance. Journal of Cell Science, 126(Pt 8), 1713–1719. 10.1242/jcs.125773 23641065 PMC3678406

[acel12943-bib-0042] Larsson, L. , Sjödin, B. , & Karlsson, J. (1978). Histochemical and biochemical changes in human skeletal muscle with age in sedentary males, age 22–65 years. Acta Physiologica Scandinavica, 103(1), 31–39. 10.1111/j.1748-1716.1978.tb06187.x 208350

[acel12943-bib-0043] Lerner, L. , Hayes, T. G. , Tao, N. , Krieger, B. , Feng, B. , Wu, Z. , … Garcia, J. M. (2015). Plasma growth differentiation factor 15 is associated with weight loss and mortality in cancer patients. Journal of Cachexia, Sarcopenia and Muscle, 6(4), 317–324. 10.1002/jcsm.12033 PMC4670740 26672741

[acel12943-bib-0044] Levine, A. J. , & Brivanlou, A. H. (2006). GDF3, a BMP inhibitor, regulates cell fate in stem cells and early embryos. Development, 133(2), 209–216. 10.1242/dev.02192 16339188

[acel12943-bib-0045] Markofski, M. M. , Dickinson, J. M. , Drummond, M. J. , Fry, C. S. , Fujita, S. , Gundermann, D. M. , … Volpi, E. (2015). Effect of age on basal muscle protein synthesis and mTORC1 signaling in a large cohort of young and older men and women. Experimental Gerontology, 65, 1–7. 10.1016/j.exger.2015.02.015 25735236 PMC4397165

[acel12943-bib-0046] Marzetti, E. , Lees, H. A. , Wohlgemuth, S. E. , & Leeuwenburgh, C. (2009). Sarcopenia of aging: Underlying cellular mechanisms and protection by calorie restriction. BioFactors, 35(1), 28–35. 10.1002/biof.5 19319843 PMC5992495

[acel12943-bib-0047] McArdle, A. , Maglara, A. , Appleton, P. , Watson, A. J. , Grierson, I. , & Jackson, M. J. (1999). Apoptosis in multinucleated skeletal muscle myotubes. Laboratory Investigation, 79(9), 1069–1076. 10496525

[acel12943-bib-0048] McLoughlin, T. J. , Smith, S. M. , DeLong, A. D. , Wang, H. , Unterman, T. G. , & Esser, K. A. (2009). FoxO1 induces apoptosis in skeletal myotubes in a DNA‐binding‐dependent manner. American Journal of Physiology. Cell Physiology, 297(3), C548–555. 10.1152/ajpcell.00502.2008 19553561 PMC2740395

[acel12943-bib-0049] McPherron, A. C. , Lawler, A. M. , & Lee, S. J. (1997). Regulation of skeletal muscle mass in mice by a new TGF‐beta superfamily member. Nature, 387(6628), 83–90. 10.1038/387083a0 9139826

[acel12943-bib-0050] Michaeloudes, C. , Sukkar, M. B. , Khorasani, N. M. , Bhavsar, P. K. , & Chung, K. F. (2011). TGF‐β regulates Nox4, MnSOD and catalase expression, and IL‐6 release in airway smooth muscle cells. American Journal of Physiology. Lung Cellular and Molecular Physiology, 300(2), L295–304. 10.1152/ajplung.00134.2010 21131394 PMC3043811

[acel12943-bib-0051] Miller, M. S. , Lekkas, P. , Braddock, J. M. , Farman, G. P. , Ballif, B. A. , Irving, T. C. , … Vigoreaux, J. O. (2008). Aging enhances indirect flight muscle fiber performance yet decreases flight ability in Drosophila. Biophysical Journal, 95(5), 2391–2401. 10.1529/biophysj.108.130005 18515368 PMC2517049

[acel12943-bib-0052] Miller, R. A. , Harrison, D. E. , Astle, C. M. , Fernandez, E. , Flurkey, K. , Han, M. , … Strong, R. (2014). Rapamycin‐mediated lifespan increase in mice is dose and sex dependent and metabolically distinct from dietary restriction. Aging Cell, 13(3), 468–477. 10.1111/acel.12194 24341993 PMC4032600

[acel12943-bib-0053] Miquel, J. , Economos, A. C. , Fleming, J. , & Johnson, J. E. (1980). Mitochondrial role in cell aging. Experimental Gerontology, 15(6), 575–591. 10.1016/0531-5565(80)90010-8 7009178

[acel12943-bib-0054] Montero, R. , Yubero, D. , Villarroya, J. , Henares, D. , Jou, C. , Rodríguez, M. A. , … Jimenez‐Mallebrera, C. (2016). GDF‐15 is elevated in children with mitochondrial diseases and is induced by mitochondrial dysfunction. PLoS ONE, 11(2), e0148709 10.1371/journal.pone.0148709 26867126 PMC4750949

[acel12943-bib-0055] Moore, A. G. , Brown, D. A. , Fairlie, W. D. , Bauskin, A. R. , Brown, P. K. , Munier, M. L. , … Breit, S. N. (2000). The transforming growth factor‐ss superfamily cytokine macrophage inhibitory cytokine‐1 is present in high concentrations in the serum of pregnant women. Journal of Clinical Endocrinology and Metabolism, 85(12), 4781–4788. 10.1210/jcem.85.12.7007 11134143

[acel12943-bib-0056] Morita, M. , Gravel, S.‐P. , Chénard, V. , Sikström, K. , Zheng, L. , Alain, T. , … Sonenberg, N. (2013). mTORC1 controls mitochondrial activity and biogenesis through 4E‐BP‐dependent translational regulation. Cell Metabolism, 18(5), 698–711. 10.1016/j.cmet.2013.10.001 24206664

[acel12943-bib-0057] Mullican, S. E. , Lin‐Schmidt, X. , Chin, C.‐N. , Chavez, J. A. , Furman, J. L. , Armstrong, A. A. , … Rangwala, S. M. (2017). GFRAL is the receptor for GDF15 and the ligand promotes weight loss in mice and nonhuman primates. Nature Medicine, 23(10), 1150–1157. 10.1038/nm.4392 28846097

[acel12943-bib-0058] Murakoshi, M. , Osamura, Y. , & Watanabe, K. (1985). Mitochondrial alterations in aged rat adrenal cortical cells. Tokai Journal of Experimental and Clinical Medicine, 10(5), 531–536. 3837404

[acel12943-bib-0059] Narici, M. V. , & Maffulli, N. (2010). Sarcopenia: Characteristics, mechanisms and functional significance. British Medical Bulletin, 95, 139–159. 10.1093/bmb/ldq008 20200012

[acel12943-bib-0060] Pistilli, E. E. , Siu, P. M. , & Alway, S. E. (2006). Molecular regulation of apoptosis in fast plantaris muscles of aged rats. Journals of Gerontology. Series A, Biological Sciences and Medical Sciences, 61(3), 245–255. 10.1093/gerona/61.3.245 PMC2778222 16567372

[acel12943-bib-0061] Sabatini, D. M. (2017). Twenty‐five years of mTOR: Uncovering the link from nutrients to growth. Proceedings of the National Academy of Sciences of the United States of America, 114(45), 11818–11825. 10.1073/pnas.1716173114 29078414 PMC5692607

[acel12943-bib-0062] Saxton, R. A. , & Sabatini, D. M. (2017). mTOR Signaling in Growth, Metabolism, and Disease. Cell, 169(2), 361–371. 10.1016/j.cell.2017.03.035 28388417

[acel12943-bib-0063] Schieke, S. M. , Phillips, D. , McCoy, J. P. , Aponte, A. M. , Shen, R. F. , Balaban, R. S. , & Finkel, T. (2006). The mammalian target of rapamycin (mTOR) pathway regulates mitochondrial oxygen consumption and oxidative capacity. Journal of Biological Chemistry, 281(37), 27643–27652. 10.1074/jbc.M603536200 16847060

[acel12943-bib-0064] Short, K. R. , Bigelow, M. L. , Kahl, J. , Singh, R. , Coenen‐Schimke, J. , Raghavakaimal, S. , & Nair, K. S. (2005). Decline in skeletal muscle mitochondrial function with aging in humans. Proceedings of the National Academy of Sciences of the United States of America, 102(15), 5618–5623. 10.1073/pnas.0501559102 15800038 PMC556267

[acel12943-bib-0065] Shukla, S. , Rizvi, F. , Raisuddin, S. , & Kakkar, P. (2014). FoxO proteins' nuclear retention and BH3‐only protein Bim induction evoke mitochondrial dysfunction‐mediated apoptosis in berberine‐treated HepG2 cells. Free Radical Biology and Medicine, 76, 185–199. 10.1016/j.freeradbiomed.2014.07.039 25128467

[acel12943-bib-0066] Stephen , A., Michael , R. M., & Parco , S. (2011). Aging and apoptosis in muscle (7th ed.). San Diego, CA: Academic Press.

[acel12943-bib-0067] Tandler, B. , & Hoppel, C. L. (1986). Studies on giant mitochondria. Annals of the New York Academy of Sciences, 488, 65–81. 10.1111/j.1749-6632.1986.tb46548.x 3555262

[acel12943-bib-0068] Tang, H. , Inoki, K. , Lee, M. , Wright, E. , Khuong, A. , Khuong, A. , … Shrager, J. B. (2014). mTORC1 promotes denervation‐induced muscle atrophy through a mechanism involving the activation of FoxO and E3 ubiquitin ligases. Science Signalling, 7(314), ra18 10.1126/scisignal.2004809 24570486

[acel12943-bib-0069] Tsai, V. W. , Lin, S. , Brown, D. A. , Salis, A. , & Breit, S. N. (2016). Anorexia‐cachexia and obesity treatment may be two sides of the same coin: Role of the TGF‐b superfamily cytokine MIC‐1/GDF15. International Journal of Obesity, 40(2), 193–197. 10.1038/ijo.2015.242 26620888

[acel12943-bib-0070] Tsai, V.‐W. , Macia, L. , Johnen, H. , Kuffner, T. , Manadhar, R. , Jørgensen, S. B. , … Breit, S. N. (2013). TGF‐b superfamily cytokine MIC‐1/GDF15 is a physiological appetite and body weight regulator. PLoS ONE, 8(2), e55174 10.1371/journal.pone.0055174 23468844 PMC3585300

[acel12943-bib-0071] Tsai, V.‐W. , Manandhar, R. , Jørgensen, S. B. , Lee‐Ng, K. K. M. , Zhang, H. P. , Marquis, C. P. , … Breit, S. N. (2014). The anorectic actions of the TGFβ cytokine MIC‐1/GDF15 require an intact brainstem area postrema and nucleus of the solitary tract. PLoS ONE, 9(6), e100370 10.1371/journal.pone.0100370 24971956 PMC4074070

[acel12943-bib-0072] Villars, F. O. , Pietra, C. , Giuliano, C. , Lutz, T. A. , & Riediger, T. (2017). Oral treatment with the ghrelin receptor agonist HM01 attenuates cachexia in mice bearing colon‐26 (C26) tumors. International Journal of Molecular Sciences, 18(5), 10.3390/ijms18050986 PMC5454899 28475119

[acel12943-bib-0073] Wan, M. , Wu, X. , Guan, K. L. , Han, M. , Zhuang, Y. , & Xu, T. (2006). Muscle atrophy in transgenic mice expressing a human TSC1 transgene. FEBS Letters, 580(24), 5621–5627. 10.1016/j.febslet.2006.09.008 16996505

[acel12943-bib-0074] Weide, B. , Schäfer, T. , Martens, A. , Kuzkina, A. , Uder, L. , Noor, S. , … Wischhusen, J. (2016). High GDF‐15 serum levels independently correlate with poorer overall survival of patients with tumor‐free stage III and unresectable stage IV melanoma. The Journal of Investigative Dermatology, 136(12), 2444–2452. 10.1016/j.jid.2016.07.016 27705749

[acel12943-bib-0075] Wen, Z. , Zhong, Z. , & Darnell, J. E. (1995). Maximal activation of transcription by Stat1 and Stat3 requires both tyrosine and serine phosphorylation. Cell, 82(2), 241–250. 7543024 10.1016/0092-8674(95)90311-9

[acel12943-bib-0076] Whitman, S. A. , Wacker, M. J. , Richmond, S. R. , & Godard, M. P. (2005). Contributions of the ubiquitin‐proteasome pathway and apoptosis to human skeletal muscle wasting with age. Pflügers Archiv ‐ European Journal of Physiology, 450(6), 437–446. 10.1007/s00424-005-1473-8 15952031

[acel12943-bib-0077] Wiklund, F. E. , Bennet, A. M. , Magnusson, P. K. E. , Eriksson, U. K. , Lindmark, F. , Wu, L. , … Brown, D. A. (2010). Macrophage inhibitory cytokine‐1 (MIC‐1/GDF15): A new marker of all‐cause mortality. Aging Cell, 9(6), 1057–1064. 10.1111/j.1474-9726.2010.00629.x 20854422 PMC4139960

[acel12943-bib-0078] Wildey, G. M. , Patil, S. , & Howe, P. H. (2003). Smad3 potentiates transforming growth factor beta (TGFbeta )‐induced apoptosis and expression of the BH3‐only protein Bim in WEHI 231 B lymphocytes. Journal of Biological Chemistry, 278(20), 18069–18077. 10.1074/jbc.M211958200 12637528

[acel12943-bib-0079] Yang, L. , Chang, C.‐C. , Sun, Z. , Madsen, D. , Zhu, H. , Padkjær, S. B. , … Jørgensen, S. B. (2017). GFRAL is the receptor for GDF15 and is required for the anti‐obesity effects of the ligand. Nature Medicine, 23(10), 1158–1166. 10.1038/nm.4394 28846099

[acel12943-bib-0080] Yatsuga, S. , Fujita, Y. , Ishii, A. , Fukumoto, Y. , Arahata, H. , Kakuma, T. , … Koga, Y. (2015). Growth differentiation factor 15 as a useful biomarker for mitochondrial disorders. Annals of Neurology, 78(5), 814–823. 10.1002/ana.24506 26463265 PMC5057301

[acel12943-bib-0081] Yokogami, K. , Wakisaka, S. , Avruch, J. , & Reeves, S. A. (2000). Serine phosphorylation and maximal activation of STAT3 during CNTF signaling is mediated by the rapamycin target mTOR. Current Biology, 10(1), 47–50. 10.1016/S0960-9822(99)00268-7 10660304

[acel12943-bib-0082] Zimmers, T. A. , Jin, X. , Hsiao, E. C. , McGrath, S. A. , Esquela, A. F. , & Koniaris, L. G. (2005). Growth differentiation factor‐15/macrophage inhibitory cytokine‐1 induction after kidney and lung injury. Shock, 23(6), 543–548. 15897808

