# Supplementary material for: mTORC1 underlies age‐related muscle fiber damage and loss by inducing oxidative stress and catabolism
Source: Aging Cell. 2019 Mar 29;18(3):e12943. doi: 10.1111/acel.12943 (PMC6516169; doi:10.1111/acel.12943)
Supplement: Supplementary file 1 [file ACEL-18-e12943-s001.pdf]

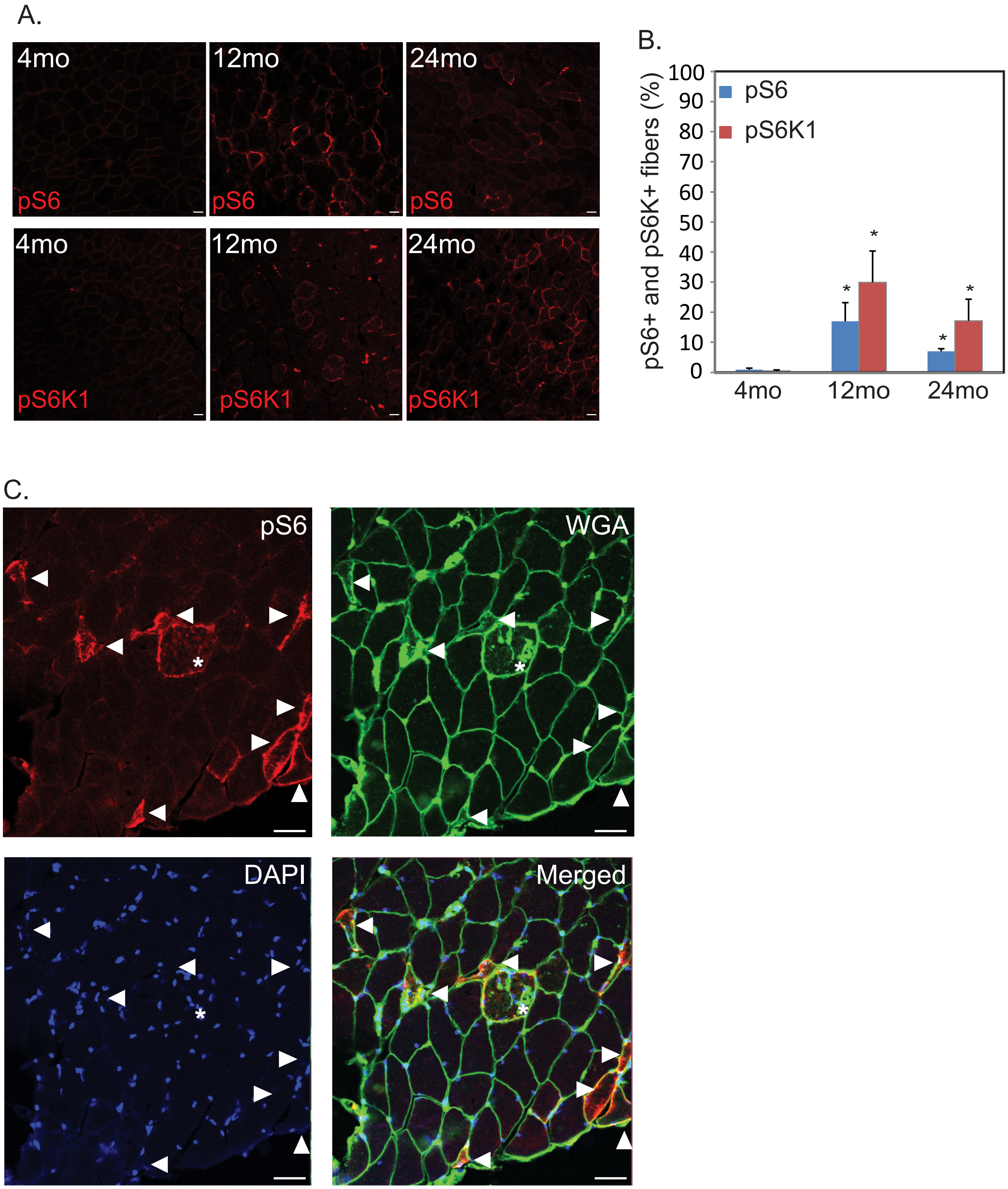
A.
4mo
12mo
24mo
pS6
pS6
pS6
4mo
12mo
24mo
pS6K1
pS6K1
pS6K1
B.
pS6+ and pS6K+ fibers (%)
100
90
80
70
60
50
40
30
20
10
0
pS6
pS6K1
4mo
12mo
24mo
C.
pS6
WGA
DAPI
Merged

Fig. S1

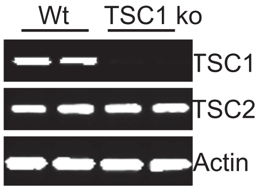

Primers:

MOUSE TSC1 Forward: GTCAATGAGCTGTACCTGGA
MOUSE TSC1 Reverse: CTTTGGTTCTGCTGGAGAAG

MOUSE TSC2 Forward: TCTACAATGATTCTGGTGAGG
MOUSE TSC2 Reverse: TGCACTGCAGGGTCAATAGGTT

Actin-gamma Forward: ACCCAGGCATTGCTGACAGGATGC
Actin-gamma Reverse: CCATCTAGAAGCATTTGCGGTGGACG

Fig. S2

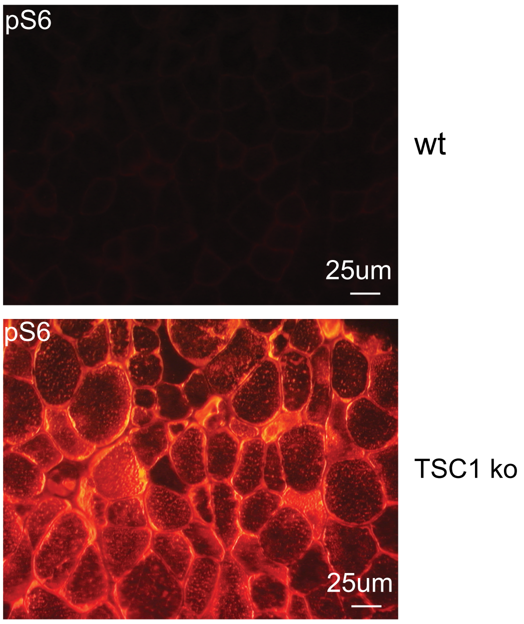

Fig. S3

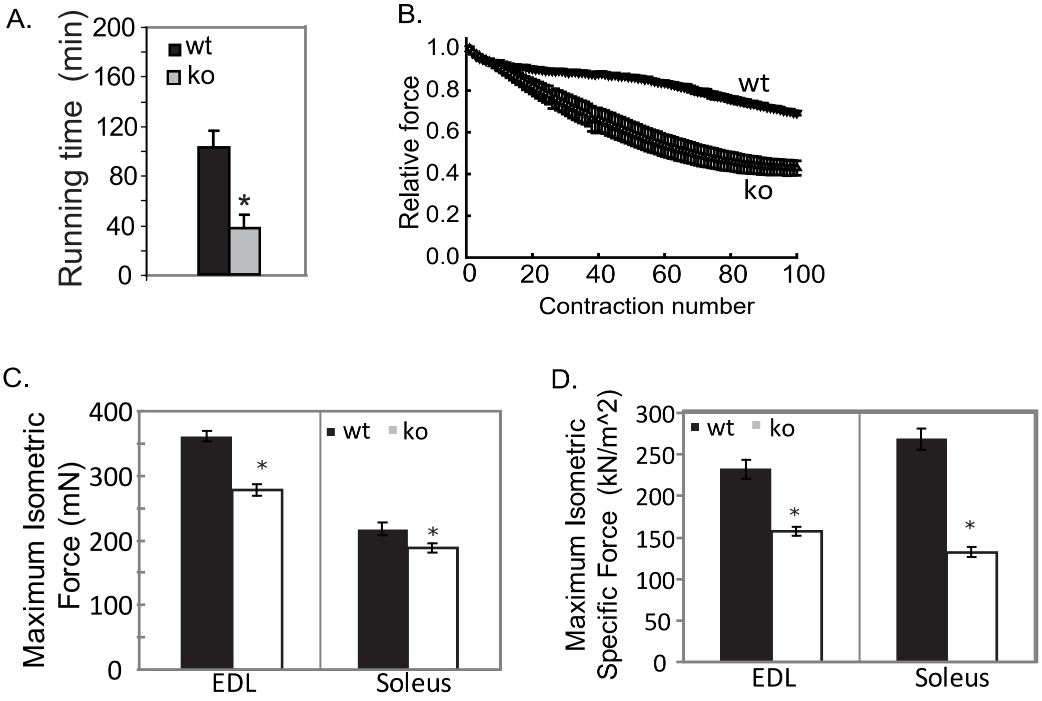

E.

The lifespan of wt and TSC1ko mice included in the survival rate calculation

	Age	**11m**	**12m**	**13m**	**14m**	**15m**	**16m**	**17m**	**18m**
**TSC1 ko**	Dead (n)	2	2		3	4		2	7
	Total (n)	33							
	Survival rate	0.94	0.88		0.79	0.67		0.61	0.39
	Comments	Found dead	Found dead		Found dead	Found dead		Feeble and euthanized	Feeble and euthanized
**Wt**	Dead (n)	0	0	0	0	0	0	0	0
	Total (n)	22							
	Survival rate	1	1	1	1	1	1	1	1

Fig. S4

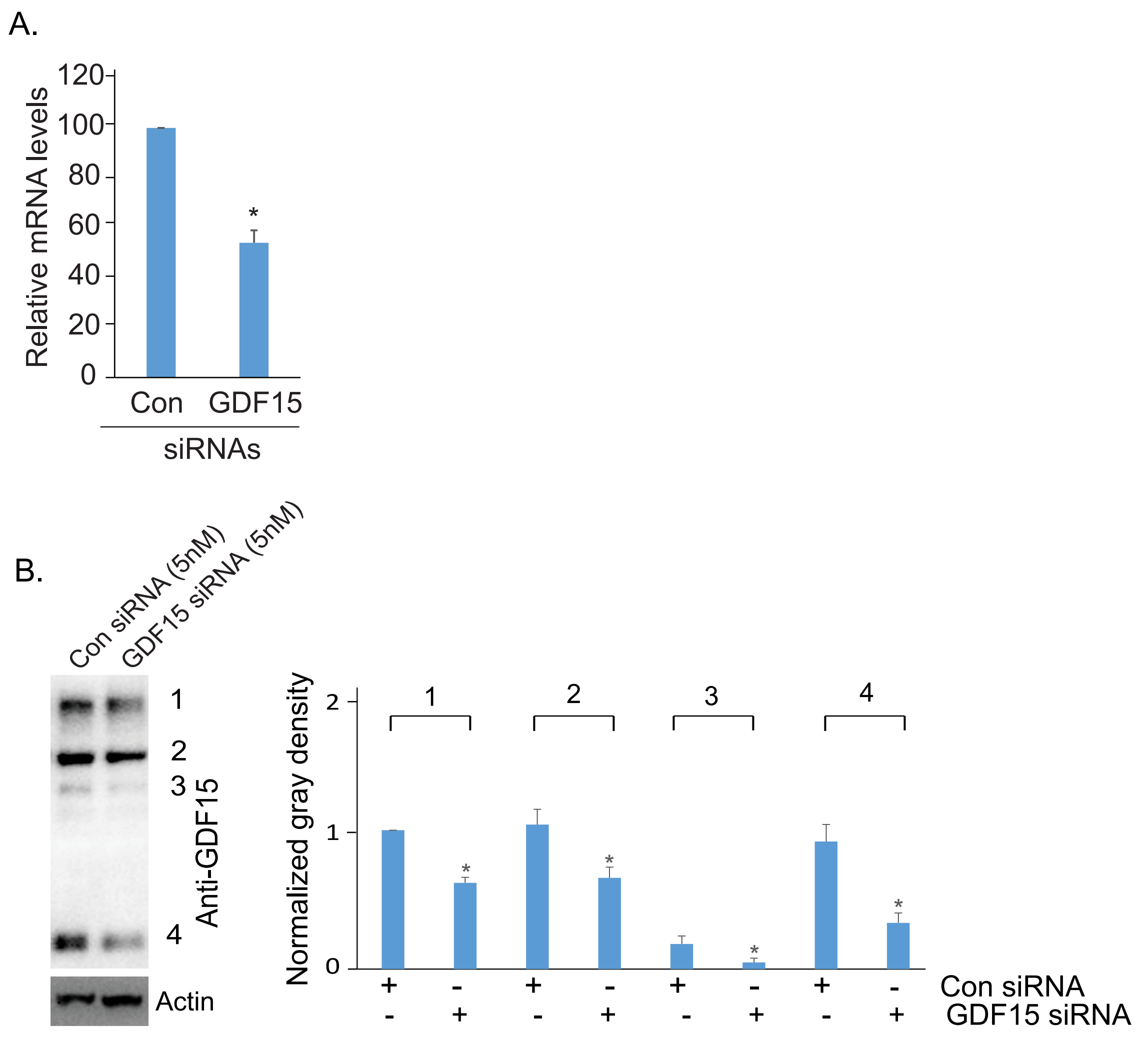

GDF15 and control siRNAs, and GDF15 anitbody were purchased from Santa Cruz Biotechnogy. C2C12 cells were transfected and lysates were used for RNA and protein analysis.

PCR primers used :
mouse GDF15 Forward: AAGCGACATGGCCCCG
mouse GDF15 reverse: CAATCTCACCTCTGGACTGAGTA

Fig. S5

C2C12 myotubes:

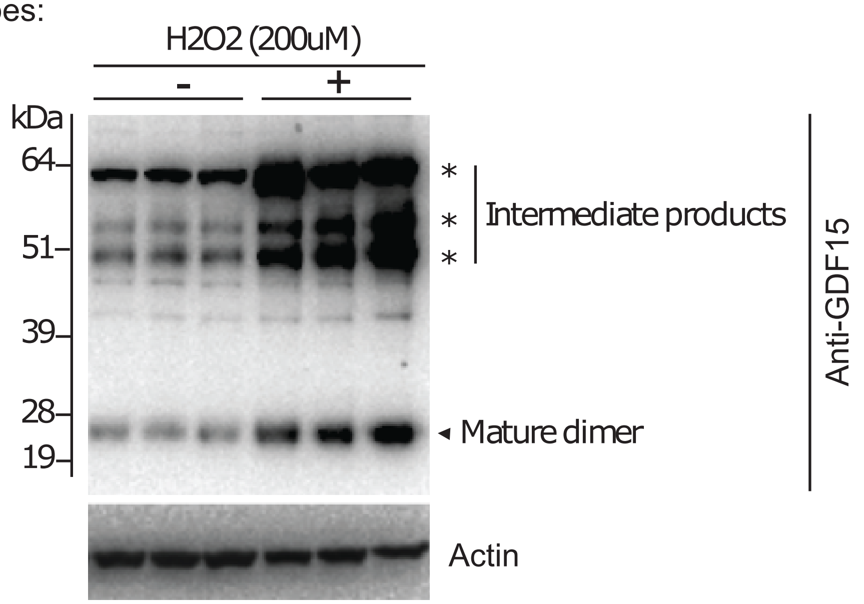

Explanation of GDF15's bands detected by Western blot:

Besides the mature dimer, primary GDF15 protein yields several intermediate products during the maturation process, which include the full-length primary protein, the primary protein without signal peptide (dimer and monomer), and these intermediate products that are posttranslationally modified by N-linked glycosylation, as shown in the following diagram. The presence of these intermediate products may vary depending on the cellular status. These explain the presence of multiple detected bands by western blot

GDF15 protein:

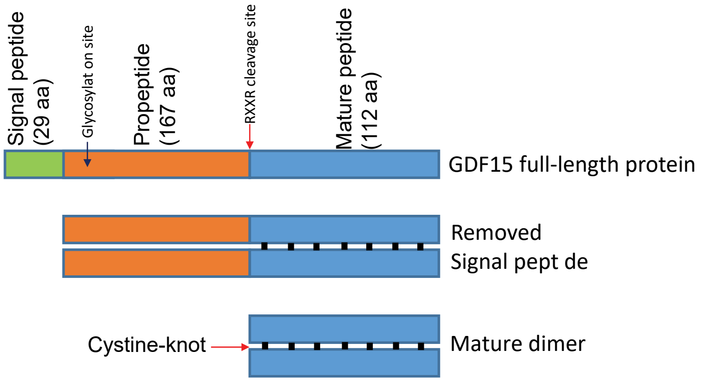

(Adapted from http://atlasgeneticsoncology.org/Genes/GDF15ID40701ch19p13.html)

Fig. S6

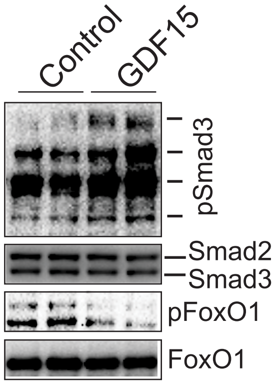

Fig. S7 Cultured C2C12 cells transfected with GDF15-expressing plasmid or control plasmid. Totoal proteins were extacted and subjected to western blot analysis

Fig. 7A

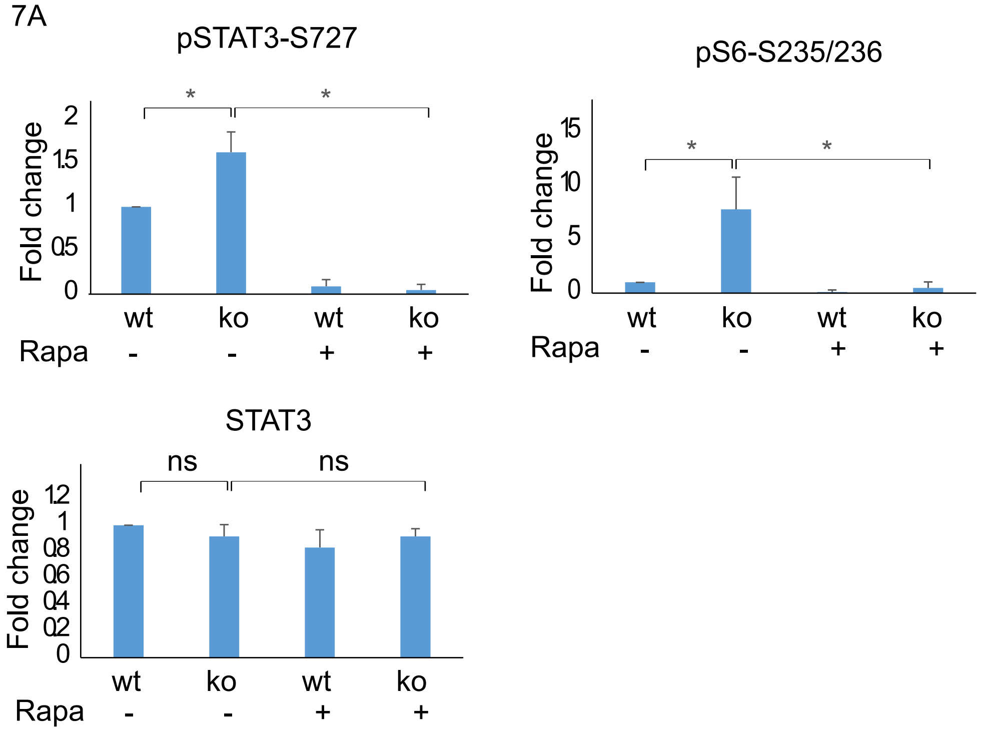

Fig. 7D

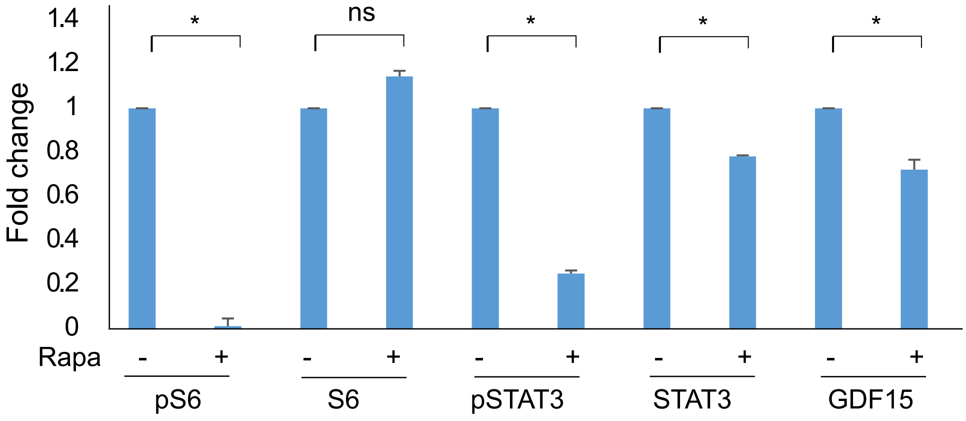

Fig. 7E

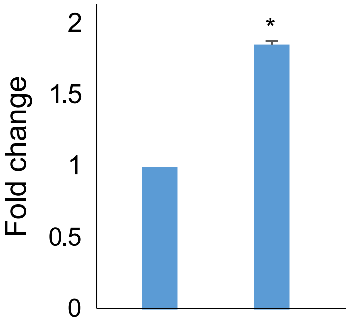

Fig. 7F

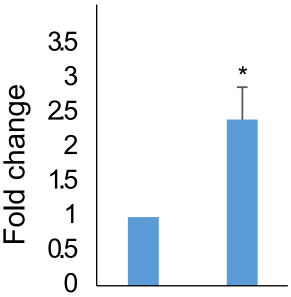

Fig. 7G

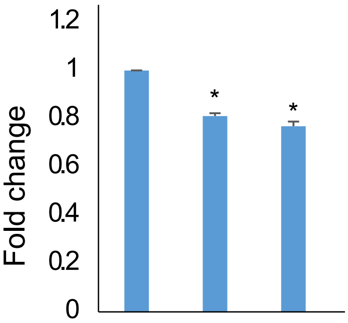

Fig. S8

A.

Patient information

Age	Sex
23	F
31	M
45	M
49	M
49	M
56	M
56	M
59	M
65	M
68	M
69	M
72	F
75	M
77	M

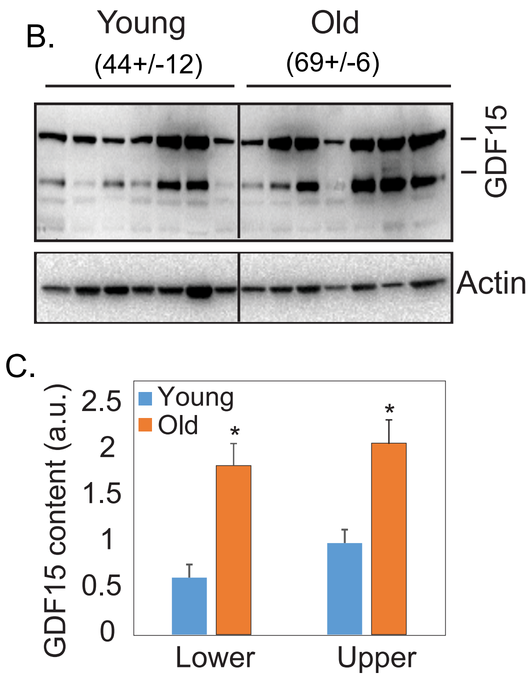

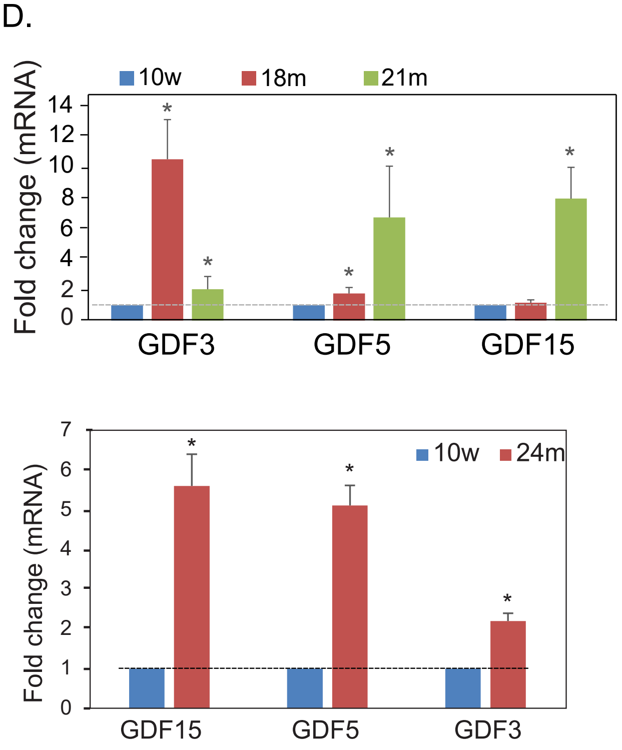

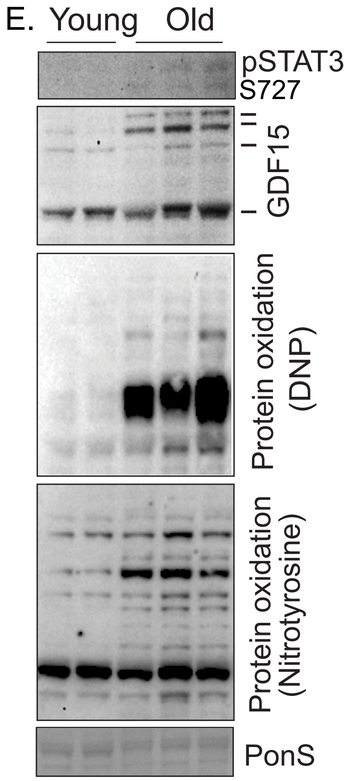

Fig. S9

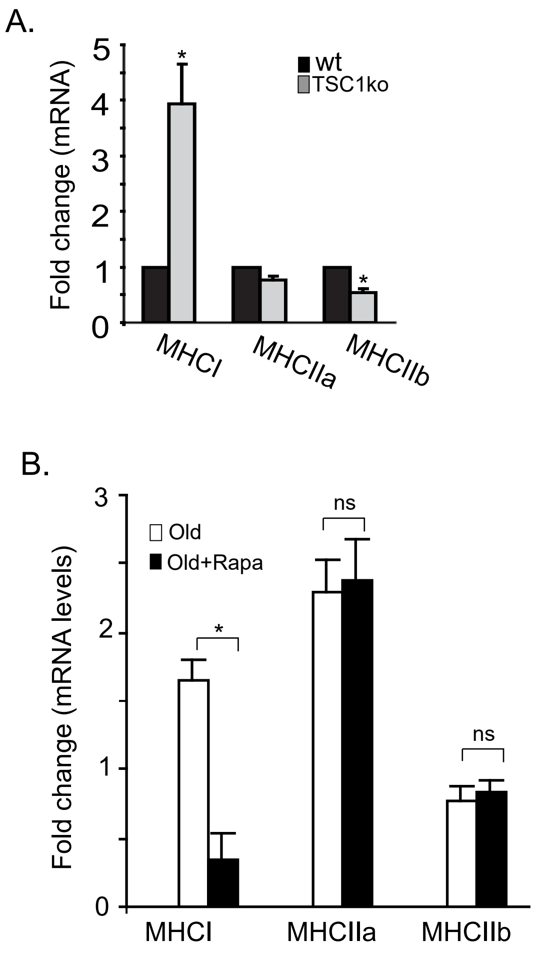
A.
Fold change (mRNA)
5
4
3
2
1
0
wt
TSC1ko
*
*
MHCI
MHCIIa
MHCIIb
B.
Fold change (mRNA levels)
3
2
1
0
Old
Old+Rapa
*
ns
ns
MHCI
MHCIIa
MHCIIb

Fig. S10

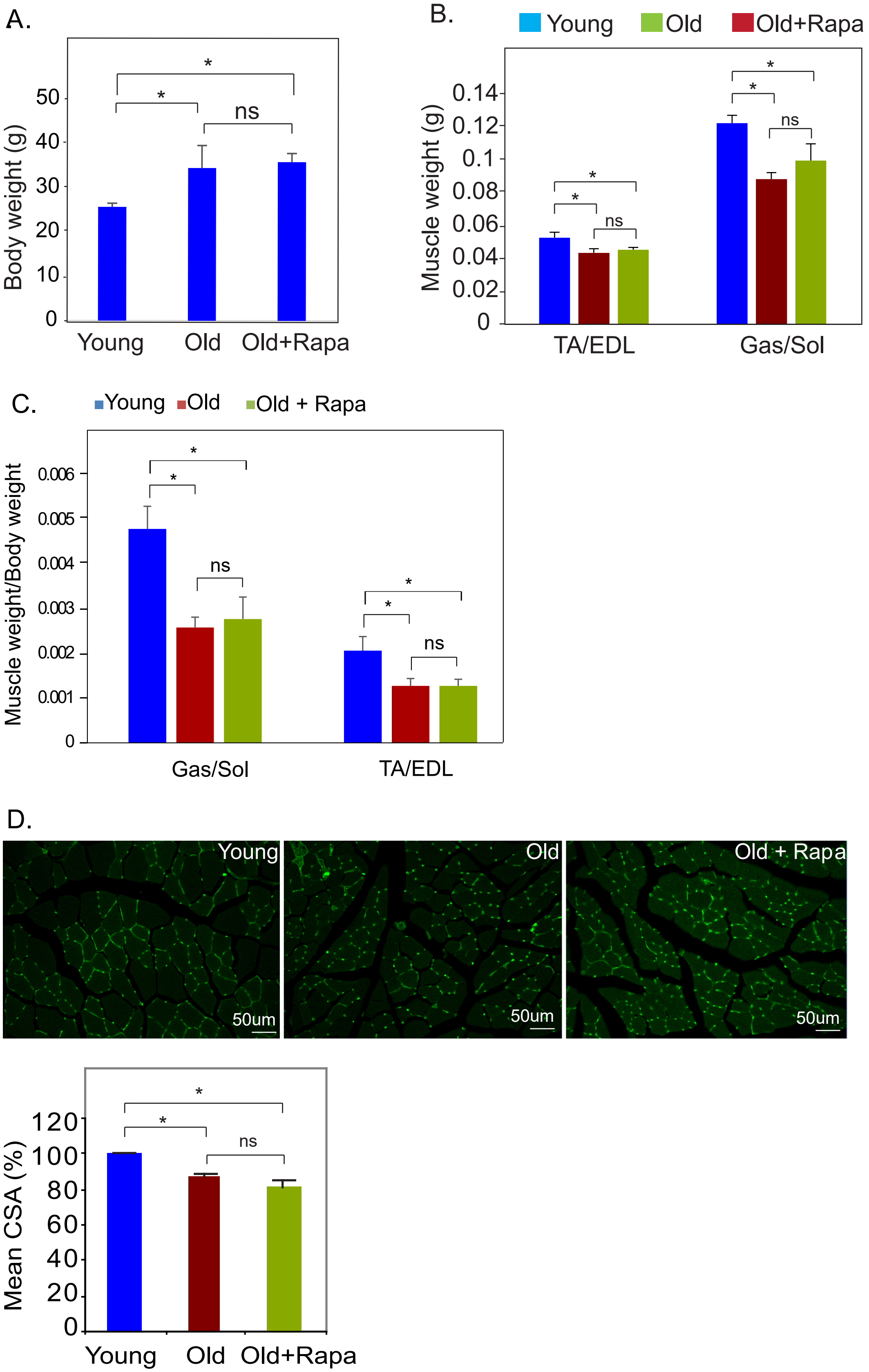
A.
Body weight (g)
0
10
20
30
40
50
*
*
ns
Young
Old
Old+Rapa
B.
Young
Old
Old+Rapa
Muscle weight (g)
0
0.02
0.04
0.06
0.08
0.1
0.12
0.14
*
*
ns
TA/EDL
Gas/Sol
C.
Young
Old
Old + Rapa
Muscle weight/Body weight
0
0.001
0.002
0.003
0.004
0.005
0.006
ns
Gas/Sol
TA/EDL
D.
Young
Old
Old + Rapa
50um
Mean CSA (%)
0
20
40
60
80
100
120
Young
Old
Old+Rapa

Fig. S11

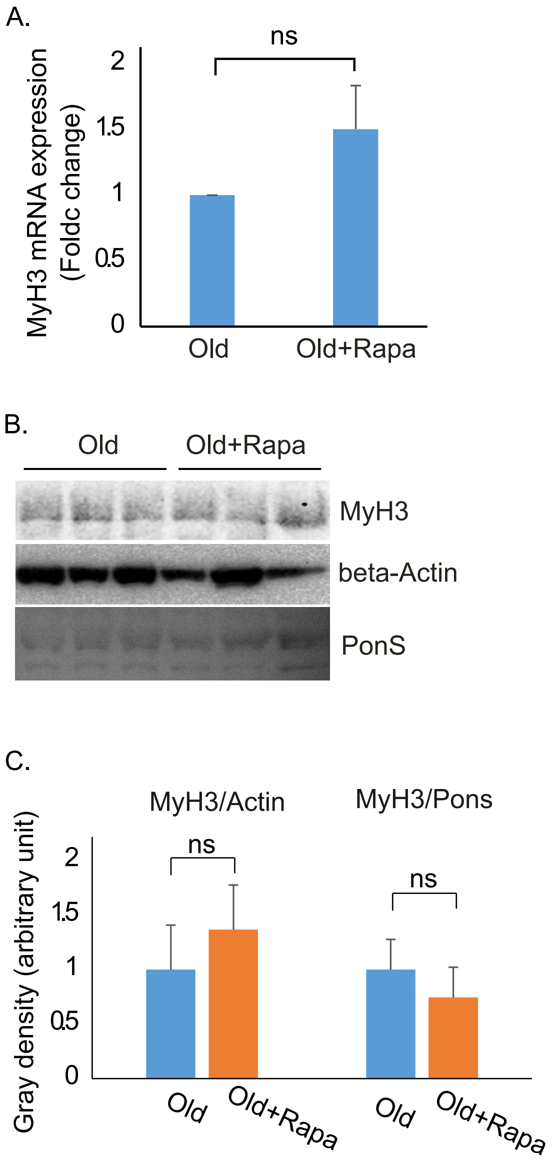
A.
ns
MyH3 mRNA expression (Foldc change)
2
1.5
1
0.5
0
Old
Old+Rapa
B.
Old
Old+Rapa
MyH3
beta-Actin
PonS
C.
MyH3/Actin
MyH3/Pons
ns
ns
Gray density (arbitrary unit)
2
1.5
1
0.5
0
Old
Old+Rapa
Old
Old+Rapa

Fig. S12

A. GFRAL is expressed in C2C12 myotubes

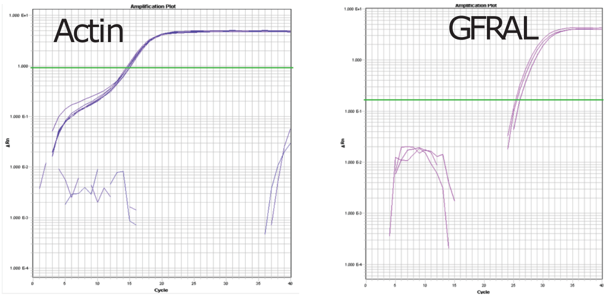

Total RNA was extracted from cultured C2C12 myotubes and used for quantitative PCR

B. GFRAL is expressed in mouse skeletal muscle tissues

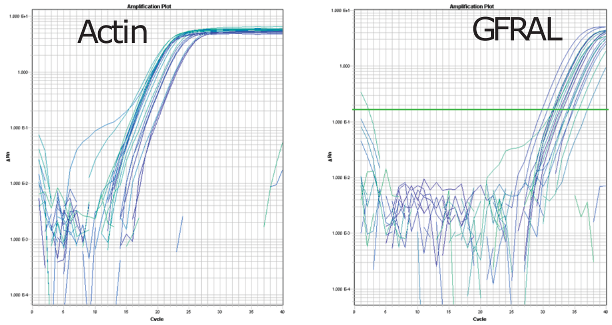

Total RNA was extracted from gastrocnemius muscle and used for quantitative PCR

C. Method fo quantitative PCR (ABI 7900HT):

Primers:

Forward: GCCTTACTCCAGGTGACTCC
Reverse: GCCTCTGTCACCTCCAAACA

Cycles:

1. 3min at 95
2. 40 cycles of 30 seconds at 95, 30 seconds at 58, 30 seconds at 72
3. 5min at 72

Fig. S13

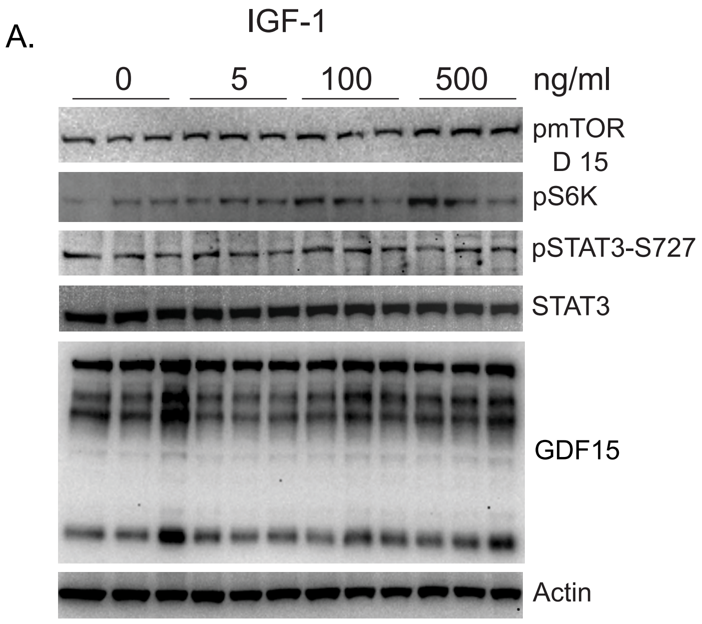

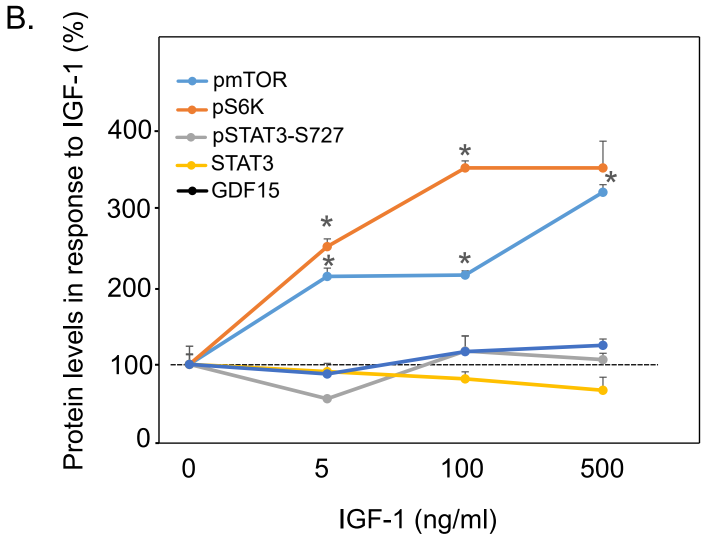

Differentiated C2C12 mytubes were treated with IGF-1 for 24 hours. Cell lysates were harvested for western blot analysis.

Fig. S14
