# Supplementary material for: mTORC1 underlies age‐related muscle fiber damage and loss by inducing oxidative stress and catabolism
Source: Aging Cell. 2019 Mar 29;18(3):e12943. doi: 10.1111/acel.12943 (PMC6516169; doi:10.1111/acel.12943)
Supplement: Supplementary file 2 [file ACEL-18-e12943-s002.pdf]

Table SI: Functional test on isolated EDL and soleus muscles

	wt-EDL Mean±SEM	ko-EDL Mean±SEM	wt-sol Mean±SEM	ko-sol Mean±SEM
**Lo (mm)**	12.18±0.11	12.22±0.12	11.88±0.16	11.74±0.16
**Lf (mm)**	5.36±0.05	5.37±0.05	8.43±0.11	8.33±0.11
**Muscle mass (mg)**	8.95±0.52	9.98±0.15	7.31±0.38	12.58±0.24 *
**muscle CSA (mm^2)**	1.57±0.08	1.75±0.02	0.81±0.03	1.42±0.02 *
**Po (mN)**	361.24±8.64	277.59±8.89 *	217.91±9.28	187.96±6.76 *
**sPo (kN/m^2)**	232.13±10.88	157.07±5.3 *	268.45±12.36	132.45±6.74 *
**Po after 5 min. protocol (mN)**	60.12±3.61	50.02±4.38	147.46±7.67	79.02±6.86 *
**Force deficit after 5 min. protocol**	0.83±0.00	0.82±0.01	0.32±0.01	0.57±0.03 *
**Po after 5 min. rest**	174.55±6.55	146.37±8.91	210.61±7.98	145.16±8.48 *
**Force deficit after 5 min. rest**	0.51±0.01	0.47±0.02	0.03±0.01	0.22±0.03 *
**% recovery**	0.38±0.02	0.42±0.02	0.9±0.04	0.62±0.04 *
**Pt (mN)**	105.53±4.13	112.5±4.45	38.43±2.32	29.43±3.06 *
**TTPT (ms)**	23.92±2.07	16.92±1.05	41.74±4.01	26.59±2.12 *
**HRT (ms)**	16.39±0.97	16.93±0.82	39.7±2.19	35.51±1.26
**Pt/Po**	0.29±0.00	0.4±0.01*	0.17±0	0.15±0.01

Lo: optimal muscle length; Lf: fiber length; CSA: cross sectin area; Po: peak tetanic tension; sPo: specific tetanic tension; Pt: peak twich tension; TTPT: time to peak tension; HRT: half relaxation time. wt (n=6), ko (n=10); * significant difference.
