# Supplementary material for: mTORC1 underlies age‐related muscle fiber damage and loss by inducing oxidative stress and catabolism
Source: Aging Cell. 2019 Mar 29;18(3):e12943. doi: 10.1111/acel.12943 (PMC6516169; doi:10.1111/acel.12943)
Supplement: Supplementary file 3 [file ACEL-18-e12943-s003.docx]

**mTORC1 Underlies Age-related Muscle Fiber Damage and Loss**

**by Inducing Oxidative Stress and Catabolism**

Huibin Tang^1,2^, Ken Inoki^3^, Susan V. Brooks^4^, Hideki Okazawa^5^, Myung Lee^1,2^, Junying Wang^3^, Michael Kim^1,2^, Catherine Kennedy^1,2^, Peter C. Macpherson^6^, Xuhuai Ji^7^, Sabrina Van Roekel^8^, Danielle A. Fraga^1,2^, Kun Wang^1,2^, Jinguo Zhu^1,2^, Yoyo Wang^1,2^, Zelton D. Sharp^9^, Richard A. Miller^8^, Thomas A. Rando^2,10^, Daniel Goldman^╪6^, Kun-Liang Guan^╪5^ and Joseph B. Shrager^╪1,2^

**Supplementary Information**

**Supplementary methods:**

**Isolated muscle function testing**

Skeletal muscle contractile properties were assessed *in vitro*. The extensor digitorum longus (EDL) and soleus muscles were isolated and removed from anesthetized mice (I.P. injection of tribromoethanol, 400 mg/kg). Muscles were placed in a horizontal bath containing buffered mammalian Ringer solution (137mM NaCl; 24mM NaHCO_3_; 11mM glucose; 5mM KCl; 2mM CaCl_2_; 1mM MgSO_4_; 1mM NaH_2_PO_4_; and 0.025mM turbocurarine chloride) maintained at 25°C and bubbled with 95% O_2_ / 5% CO_2_ to stabilize pH at 7.4. One tendon was tied to a force transducer (model BG-50, Kulite Semiconductor Products Inc.) and the other to a fixed post. Muscles were stimulated between two stainless steel plate electrodes. Stimulation voltage and then muscle length (L_o_) were adjusted to give maximum isometric twitch force (P_t_). With muscles held at L_o_, trains of 0.2 ms stimulus pulses were applied at increasing frequencies until a maximum (P_o_) was reached. Resistance to fatigue was subsequently evaluated by testing the ability of the muscles to maintain force over time. Muscles were stimulated with 100 Hz trains of 0.5 second duration, once every 5 seconds for a total of 60 contractions for EDL muscles and 100 contractions for soleus muscles. After all force measurements, muscles were blotted and weighed, and total fiber CSA was calculated by dividing the muscle mass by the product of fiber length (determined from previously established muscle length to fiber length ratios) and muscle density, 1.06 g/cm^2^. Specific P_o_ (kN/m^2^) was calculated for each muscle by dividing P_o_ by CSA.

**Treadmill test and isolated muscle functional test**

Both the wildtype and TSC1 ko mice were used in a treadmill running study (Exer-6M 6 lane Treadmill; Columbus Instruments). Three wt and three TSC^-/-^ mice were used for analysis of treadmill running time. The study protocol was approved by the University of Michigan Institutional Animal Care and Use Committee. Treadmill was set at no inclination and at the speed of 20m/min. Mice that stayed on rest grids with an electric stimulus (20 miniAmp) for 5 seconds and repeated for 3 times were considered exhausted and the running time recorded. Running time was recorded as a fatigue index for evaluating endurance.

**Supplementary figure legend**

**Fig. S1 Increased phosphorylation of S6 and S6 kinase in aging skeletal muscles.** A) Immunostaining was performed on tibialis anterior muscles from 4, 12, and 24 month-old mice. Scale bars = 25um. B) Quantitative changes of phosphorylated S6 and S6 kinases during muscle aging. n=4, *p<0.05. C) A representative, high magnification of immunostaining of tibialis anterior muscles in old mouse was shown. Red, pS6+; Green, WGA; Blue, DAPI. Note, the fiber size of the pS6+ fibers is often reduced, in angular shape (arrow heads). A regenerative fiber is seen with centrally located nuclei (asterisk). Scale bar = 25um.

**Fig. S2 Generation of TSC1 knockout mouse line.** Muscle-specific knockout mice were acquired through breeding of floxed TSC1 and MCK-Cre mouse lines. The successful knockout of TSC1 gene expression is confirmed by RT-PCR. Primers used in RT-PCR were shown. Note that TSC2 gene expression is not influenced.

**Fig. S3 Immunostaining of phosphorylated S6 protein in wt and TSC1 ko muscles.** Cryosections of TA muscles from wildtype (wt) or TSC1 ko mice at 9 months-old were stained with anti-phospho-S6 antibody. Images were taken with confocal microscope. Shown are stacked images of 10 optical sections.

**Fig. S4 Phenotypes of the TSC1 ko mice.** A) Reduced exercise capacity in TSC1 ko mice. Exercise capacity of the TSC1 ko mice and wt controls (female, 9 months old) was tested by treadmill running to exhaustion. n=6, *p<0.05. B, C &D) TSC1 ko animals exhibit reduced muscle contractile function. B) The fatiguing characteristics during repeated maximum isometric tetanic contractions of soleus muscle. The result is shown at a relative level normalized to the initial force. KO mouse muscle fatigues faster than wt mouse muscle [n=6 (wt), n=10 (ko)]. C) Maximum isometric contractile force recorded from both the fast EDL muscle and the slow soleus muscle is lower in KO mice. [n=6 (wt), n=10 (ko). *p<0.01]. (D) Maximum specific force (calculated by normalizing to the total muscle cross-sectional area) is also lower in ko mouse muscles. E) Lifespan of the TSC1 ko mice (n=33) is reduced compared to wt (n=22) mice. The comparison of the survival rate between wt and ko mice was examined by the log-rank test (implemented in STATA). The log-rank p value is p < 0.0001. The median lifespan of the TSC1 ko mice was 18 months.

**Fig S5 GDF15 siRNA reduces the mRNA levels of GDF15**. siRNA against mouse GDF15 and control siRNA were purchased from Santa Cruz Biotechnology Inc. siRNAs (20uM) were transfected into cultured C2C12 cells for 4 days. Cell lysates were harvested for RNA and protein analysis. A) Quantitative PCR was performed to measure the GDF15 mRNA level ( n=3, *p<0.05). B) Western blot analysis was performed to measure the level of GDF15 protein. Note that GDF15 siRNA reduced the levels of all of the bands recognized by GDF15 antibody (n=3, *p<0.05).

**Fig. S6 GDF15 protein detected by western blot analysis**. C2C12 myotubes were treated with H2O2 (200uM) for 24 hours. Total lysate from C2C12 myotubes were detected with anti-GDF15 antibody by Western blot analysis.

**Fig. S7 Overexpression of GDF15 increases the phosphorylation of Smad3, but decreases the phosphorylation of FoxO1.** C2C12 cells were transfected with GDF15-expressing and control plasmids, respectively. Three days later, cell lysates were collected and subjected to western blot analysis with specific antibodies.

**Fig. S8 Quantitation of protein expression levels for Fig. 7.** Gray density was quantitated by ImageJ; fold-change is shown. Actin was used as loading control ( n=3 *p<0.05).

**Fig. S9 GDF3, 5, and 15 are induced in aged muscles.** A) The information on the age and sex of the human subjects from whom the biopsies were taken. B) GDF15 is induced in aged human skeletal muscle. Protein lysates from human latissimus muscles were subjected to western blot analysis. Young (n=7, mean 44+/-12 years old), and old (n=7, mean 69+/-6 years old). C) Quantitation was performed by measuring the gray density with image J, actin protein was used as loading control ( * p<0.05). D&E) GDF is induced in aged mouse skeletal muscle. mRNA and protein were extracted from young (4 mo) and old (24 mo) gastrocnemius muscles, and subjected to quantitative PCR (D) and western blot analysis (E). D) mRNA levels of GDF15, 5 and 3 increased during aging gastrocnemius muscles (n=4 per group, *p<0.05). Results were shown with fold changes. n=4, *p<0.05. E) GDF15 protein is upregulated in old (24-month old) vs. young (4-month old) mouse gastrocnemius muscles, accompanied by induction of the levels of oxidized proteins (DNP, nitrotyrosine) and phosphorylated STAT3 (pSTAT3-S727). Equal loading of protein is shown by Ponceau S (PonS) staining of the total proteins.

**Fig. S10 mRNA expression of myosin heavy chain (MHC).** A) mRNA expression levels of MHCI, IIa, and IIb in wildtype and TSC1 ko muscles. Fold change is shown. B) mRNA expression levels of MHCI, IIa, and IIb in young mice, old, and old with rapamycin treatment. Fold change is shown (n=4, *p<0.05).

**Fig. S11 Rapamycin treatment in normal aging mice.** A) Rapamycin treatment does not affect body weight (n=4, *p=ns). B) Rapamycin treatment does not rescue the reduced muscle weight in old mice (n=4, *p=ns). C) Rapamycin treatment does not rescue the reduced the ratio of muscle weight to body weight in old mice (n=4, p=ns). D) Rapamycin treatment does not rescue the reduced muscle fiber size in old mice. Top panel: representative images stained with WGA from young, old, and old with rapamycin treatment. Lower panel: cross-sectional area was quantitated by ImageJ. All female mice were used. Young: 2 month-old; Old: 30 month-old; Old+Rapa: 30 month-old with 10 months of rapamycin treatment. n=4, p=ns).

Fig. S12 Expression of embryonic myosin heavy chain (eMHC, MyH3) is not altered in rapamycin-treated muscle. A) The mRNA levels of eMHC were examined by quantitative PCR (n=4. p=ns). B) The protein expression levels of eMHC in muscles of the old mice and old mice with rapamycin treatment. C) Gray density was quantitated with ImageJ (n=3, p=ns).

**Fig. S13 GFRAL is expressed in cultured myotubes and skeletal muscle tissue**. Quantitative PCR was performed to amplify GFRAL mRNA from cultured C2C12 myotubes (A) and mouse skeletal muscle tissues (B), with the primers and settings shown in (C). A) mRNA was extracted from differentiated C2C12 myotubes and subjected to RT-PCR. Left panel, Actin; Right panel, GFRAL. B) mRNA was extracted from mouse gastrocnemius muscle and subjected to RT-PCR. Left panel, Actin; Right panel, GFRAL. The original amplification curves were shown.

**Fig. S14 Protein expression in response to IGF treatment of C2C12 myotubes**. Cultured C2C12 cells were differentiated into myotubes for 5 days in DMEM containing 5% horse serum. Myotubes were then treated with recombinant IGF-1 (Abmgood, Inc.) at 5, 100, 500 ng/ml for 24 hours. Cell lysates were harvested for western blot analysis with specific antibodies as indicated. Representative western blots (A) and quantitative data of the gray density of each protein normalized to actin levels (B) reveal that IGF-1 treatment activates mTORC1-S6K signaling but does not regulate GDF15 or the phosphorylation of serine 727 on STAT3.

**Supplementary table**

**Table S1** Physiological parameters from skeletal muscles of TSC1 ko muscle and wt controls. n=6 (wt) and 10 (TSC1 ko), *p<0.05.
